# Supplementary material for: Furanocoumarins from Ruta chalepensis with Amebicide Activity
Source: Molecules. 2021 Jun 16;26(12):3684. doi: 10.3390/molecules26123684 (PMC8233766; doi:10.3390/molecules26123684)
Supplement: Supplementary file 1 [file molecules-26-03684-s001.zip › molecules-1243484-supplementary.pdf]

Furanocoumarins from *Ruta chalepensis* with amebicide activity

Aldo Fabio Bazaldúa-Rodríguez [1], Ramiro Quintanilla-Licea [1*], Maria Julia Verde-Star [1], Magda Elizabeth Hernández-García [2], Javier Vargas-Villarreal [3], Jesús Norberto Garza-González [4].

[1]Universidad Autónoma de Nuevo León (UANL), Facultad de Ciencias Biológicas, Laboratorio de Fitoquímica, Av. Universidad S/N, Cd. Universitaria, San Nicolás de los Garza, C.P. 66455, Nuevo León, México

[2]Centro de Investigaciones Biomédicas del Noreste (CIBIN), Laboratorio de Fisiología Celular y Molecular, Dos de abril esquina con San Luis Potosí, Monterrey, C.P. 64720, Nuevo León, México

[3]Centro de Investigaciones Biomédicas del Noreste (CIBIN), Laboratorio de Biología y Fisiología Molecular y Celular, Dos de abril esquina con San Luis Potosí, Monterrey, C.P. 64720, Nuevo León, México

[4]Universidad de Monterrey, Av. Ignacio Morones Prieto 4500 Pte., San Pedro Garza García C.P. 66238, Nuevo León, México

***Corresponding author**:
Ramiro Quintanilla-Licea
Ramiro.quintanillalc@uanl.edu.mx; Tel. ++52-81-83579479
ORCID 0000-0002-4379-6913

Spectroscopic analysis of Rutamarin **1**

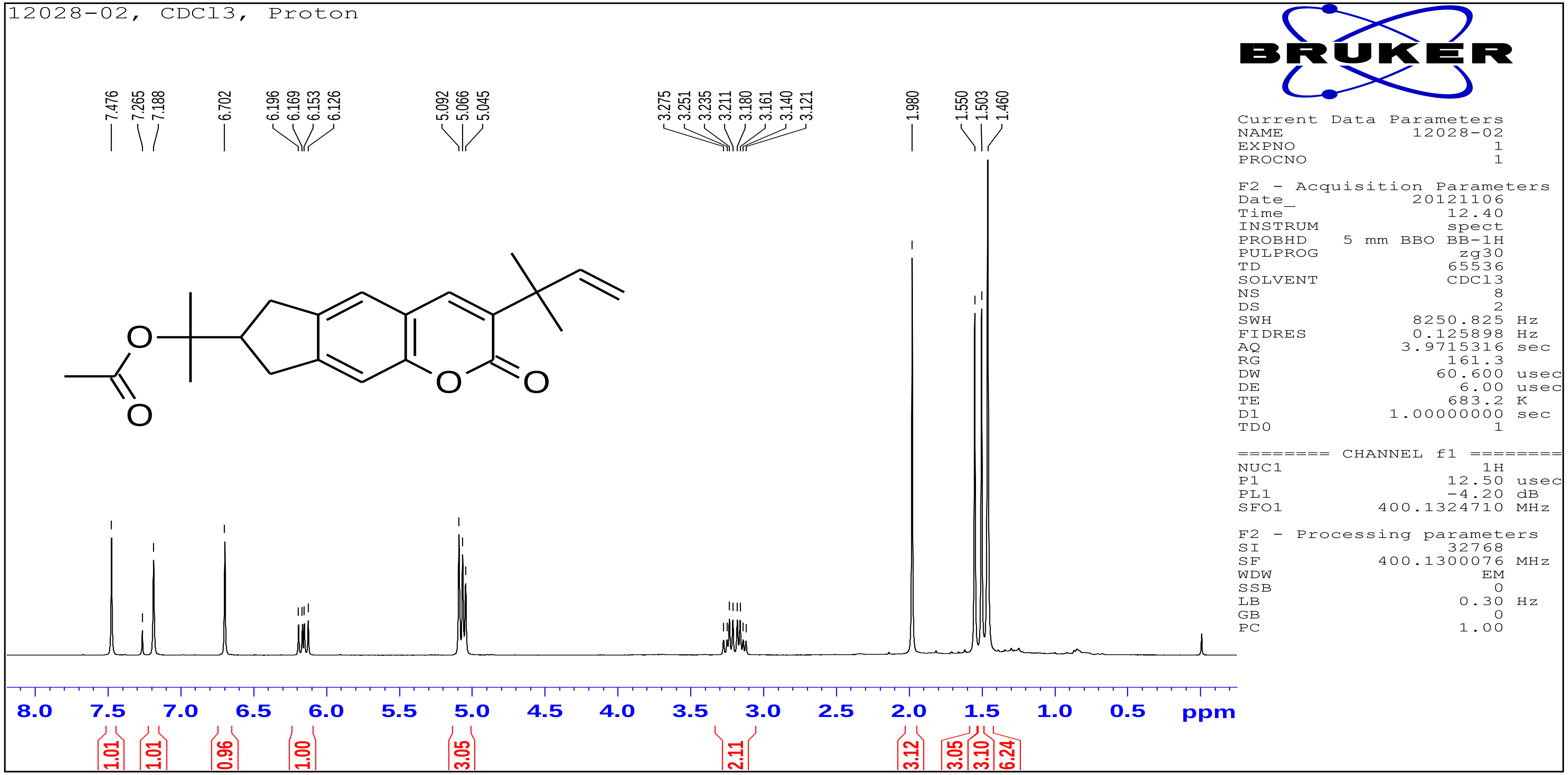

Figure S1. ^{1}H-NMR spectrum of **1**. Obtained by a Bruker Spectrometer Model Advance DPX400, 9.4 Teslas. Operating frequency 400 MHz. ($CDCl_3$).

Spectroscopic analysis of Rutamarin **1**

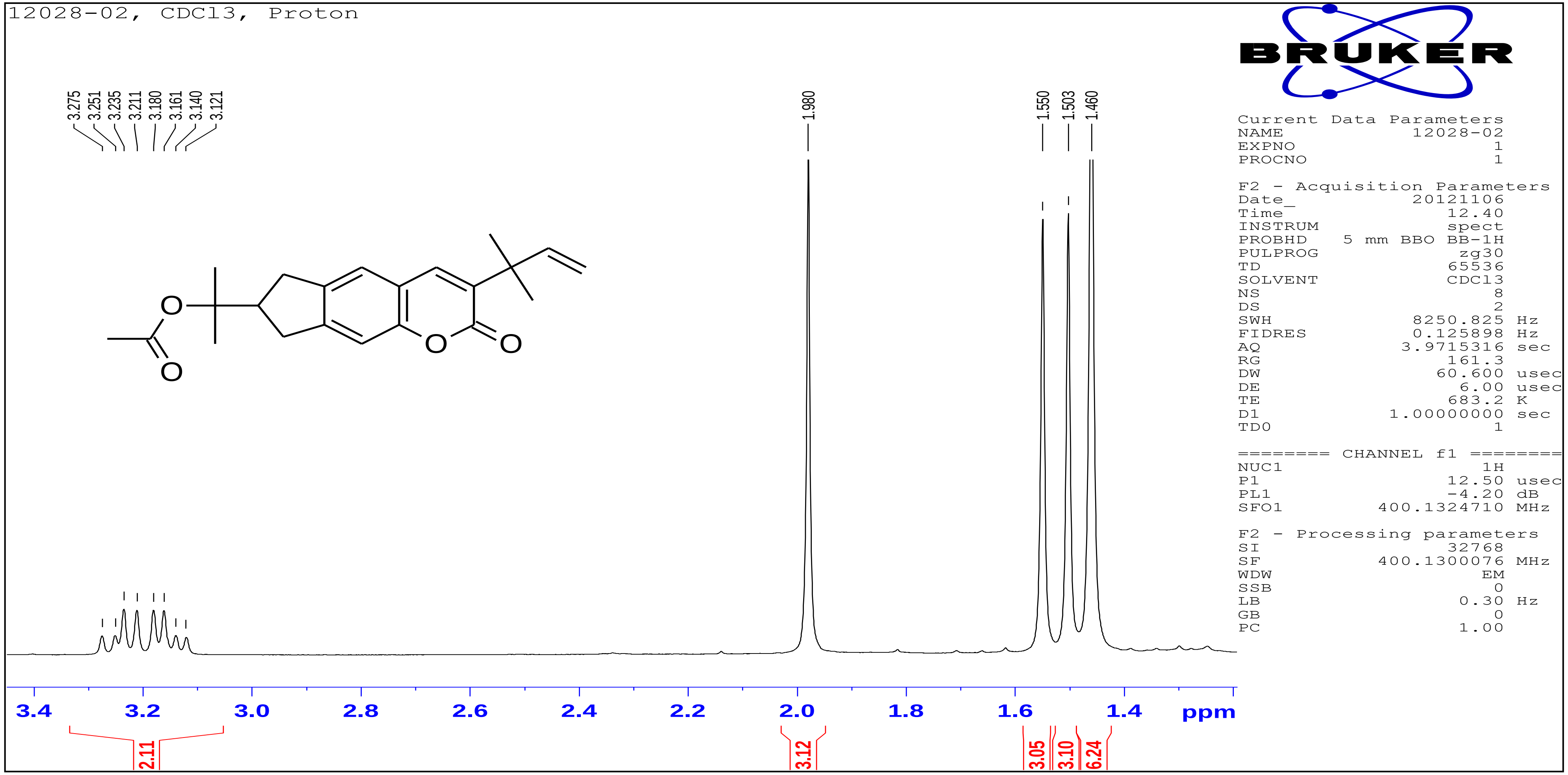

Figure S2. Expansion in the area from 1.2-3.4 ppm. ^{1}H-NMR spectrum of **1**. Obtained by a Bruker Spectrometer Model Advance DPX400, 9.4 Teslas. Operating frequency 400 MHz. ($CDCl_3$).

Spectroscopic analysis of Rutamarin **1**

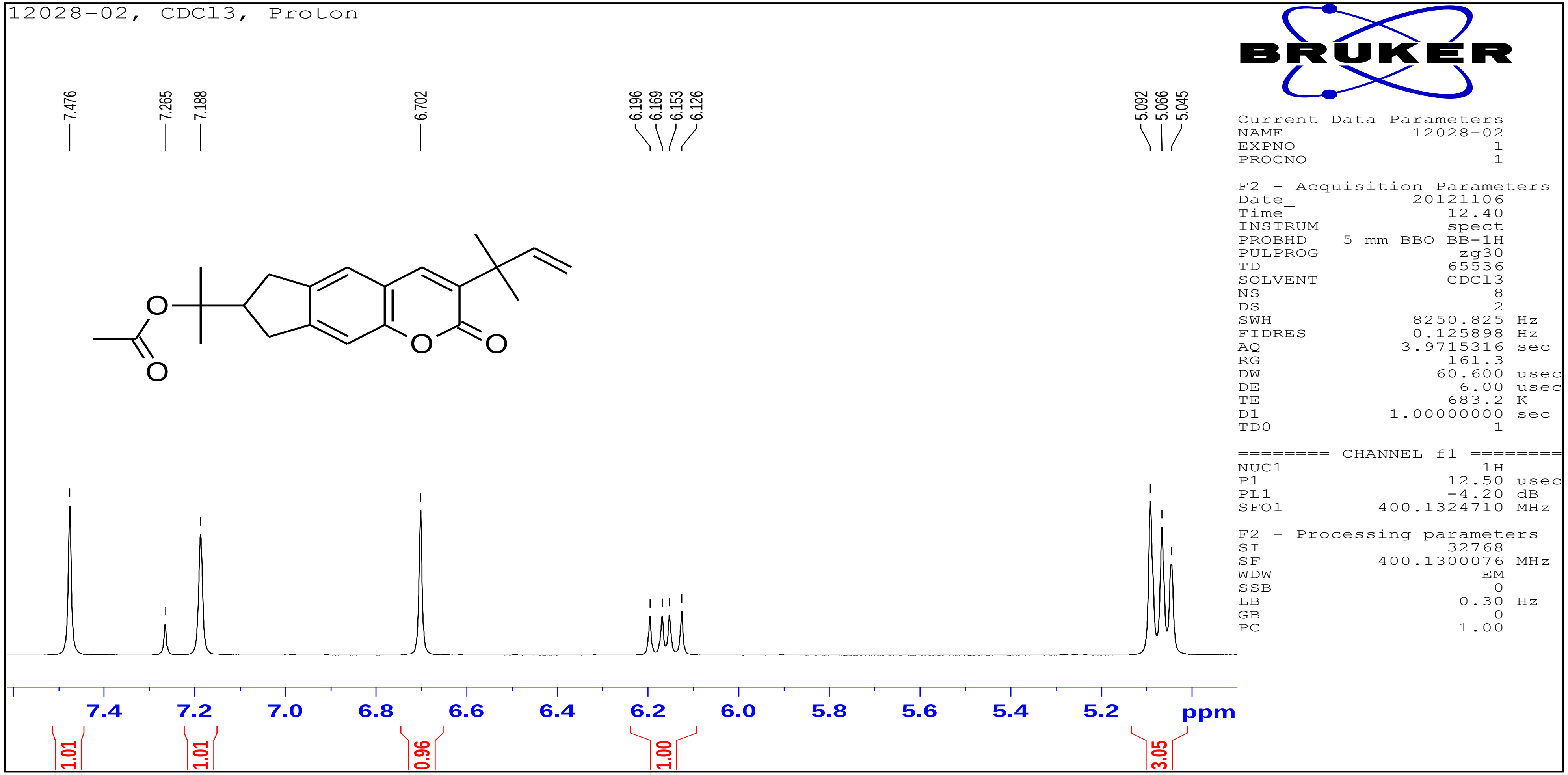

Figure S3. Expansion in the area from 4.8-7.6 ppm. ^{1}H-NMR spectrum of **1**. Obtained by a Bruker Spectrometer Model Advance DPX400, 9.4 Teslas. Operating frequency 400 MHz. ($CDCl_3$).

Spectroscopic analysis of Rutamarin **1**

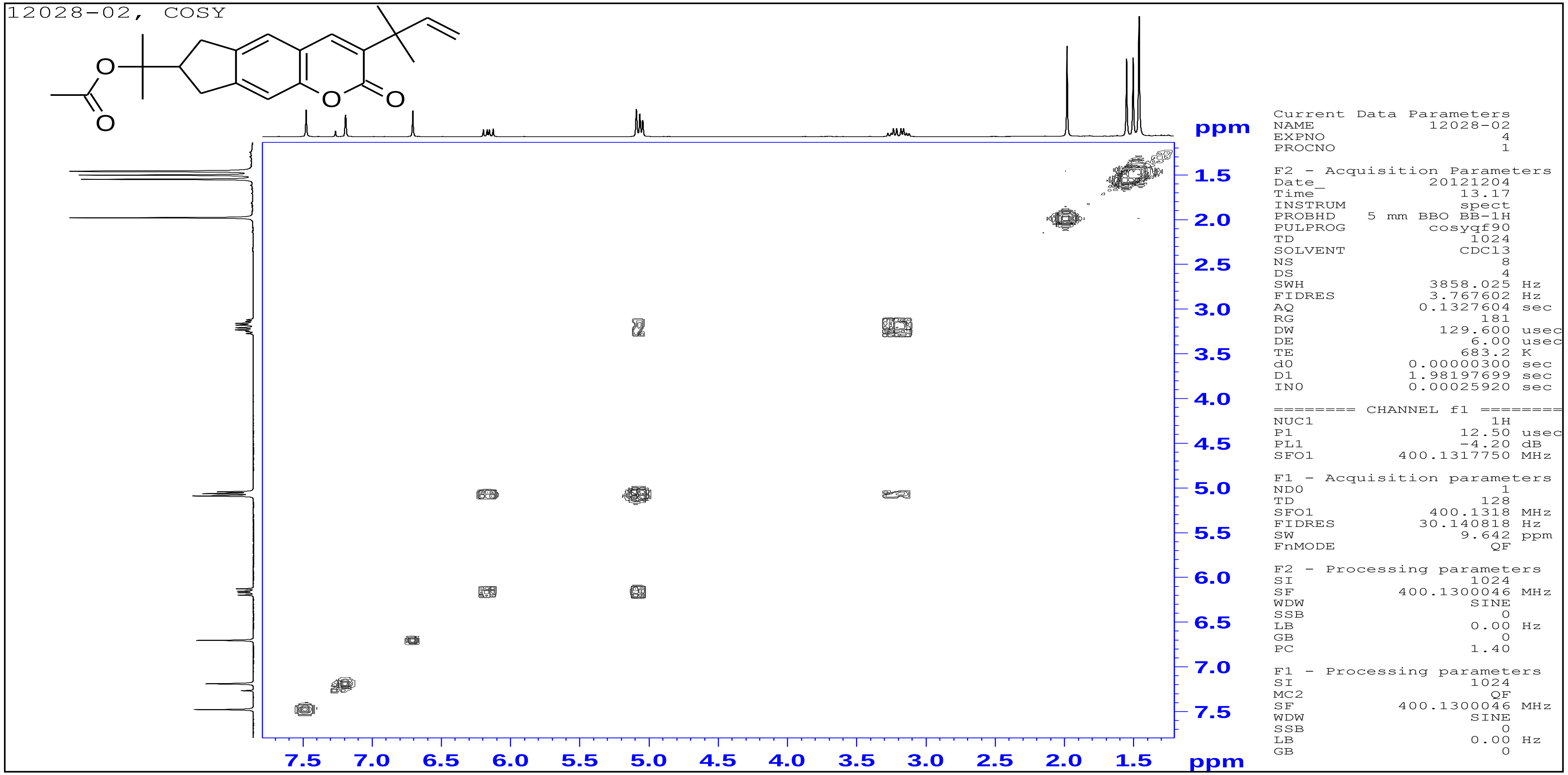

Figure S4. COSY spectrum of **1**. Obtained by a Bruker Spectrometer Model Advance DPX400, 9.4 Teslas. Operating frequency 400 MHz.($CDCl_3$).

Spectroscopic analysis of Rutamarin **1**

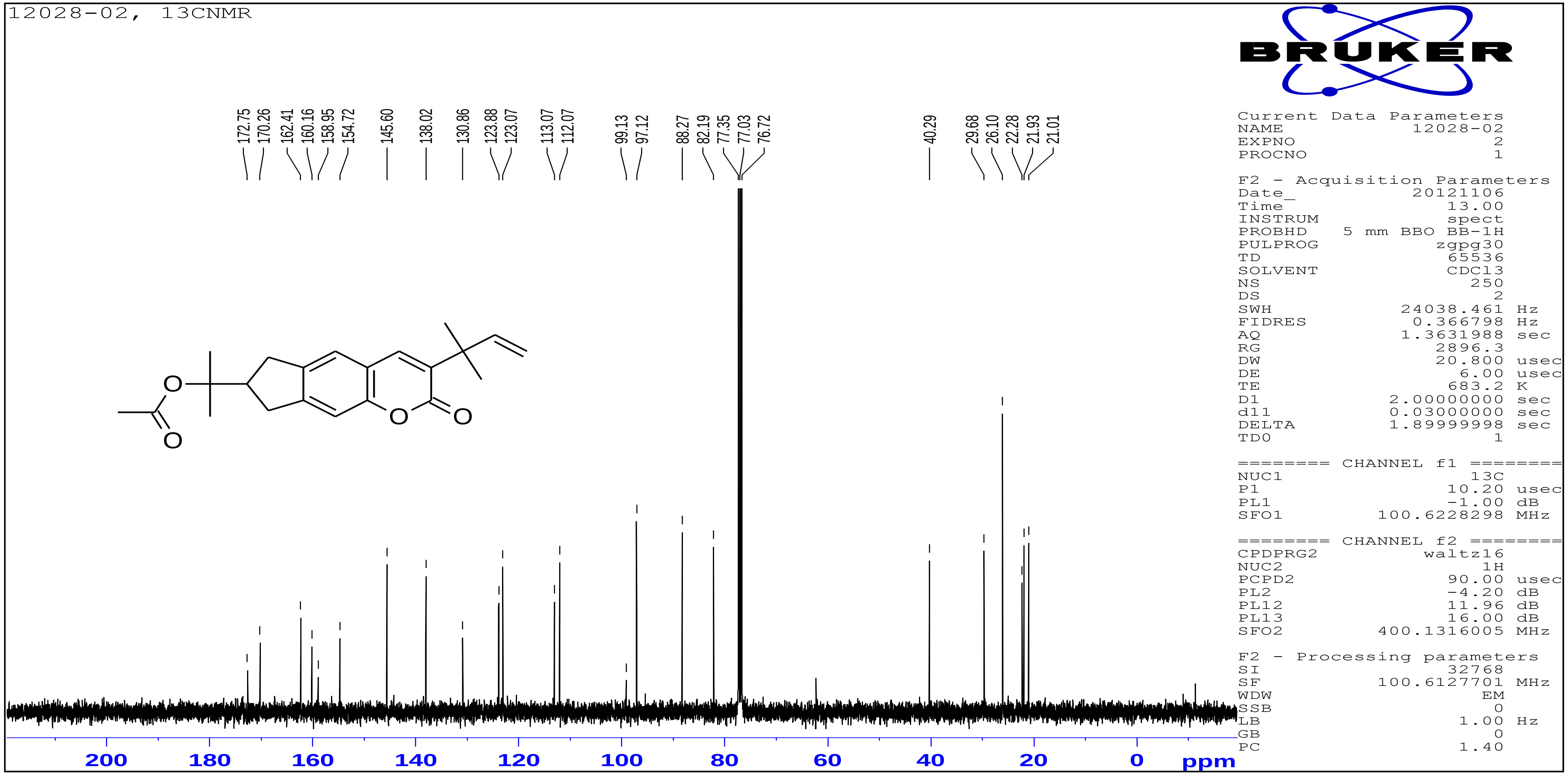

Figure S5. ^{13}C-NMR spectrum of **1**. Obtained by a Bruker Spectrometer Model Advance DPX400, 9.4 Teslas. Operating frequency 100 MHz. ($CDCl_3$).

Spectroscopic analysis of Rutamarin **1**

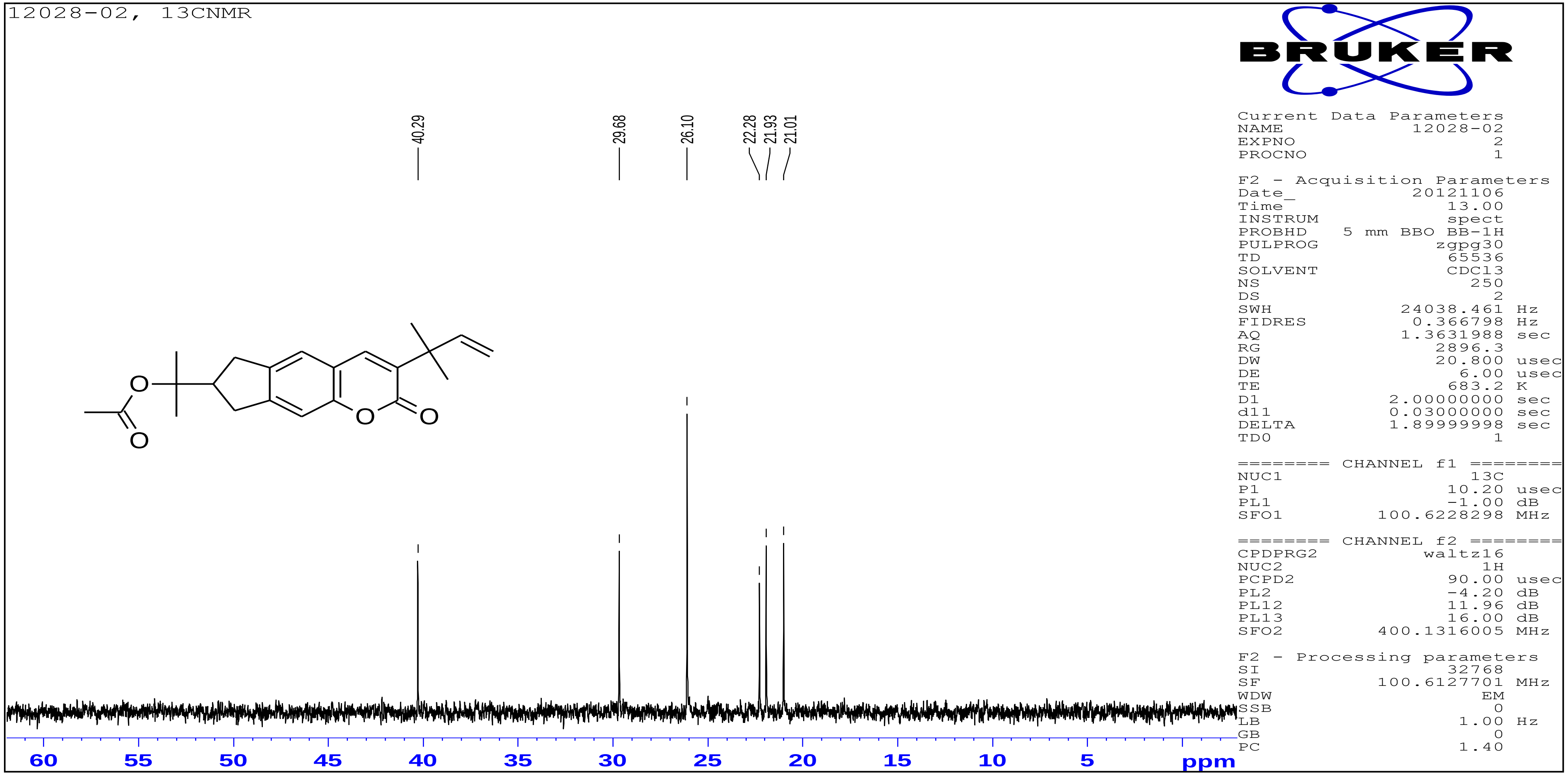

Figure S6. Expansion in the area from 0.5-60 ppm ^{13}C-NMR spectrum of **1**. Obtained by a Bruker Spectrometer Model Advance DPX400, 9.4 Teslas. Operating frequency 100 MHz. ($CDCl_3$).

Spectroscopic analysis of Rutamarin **1**

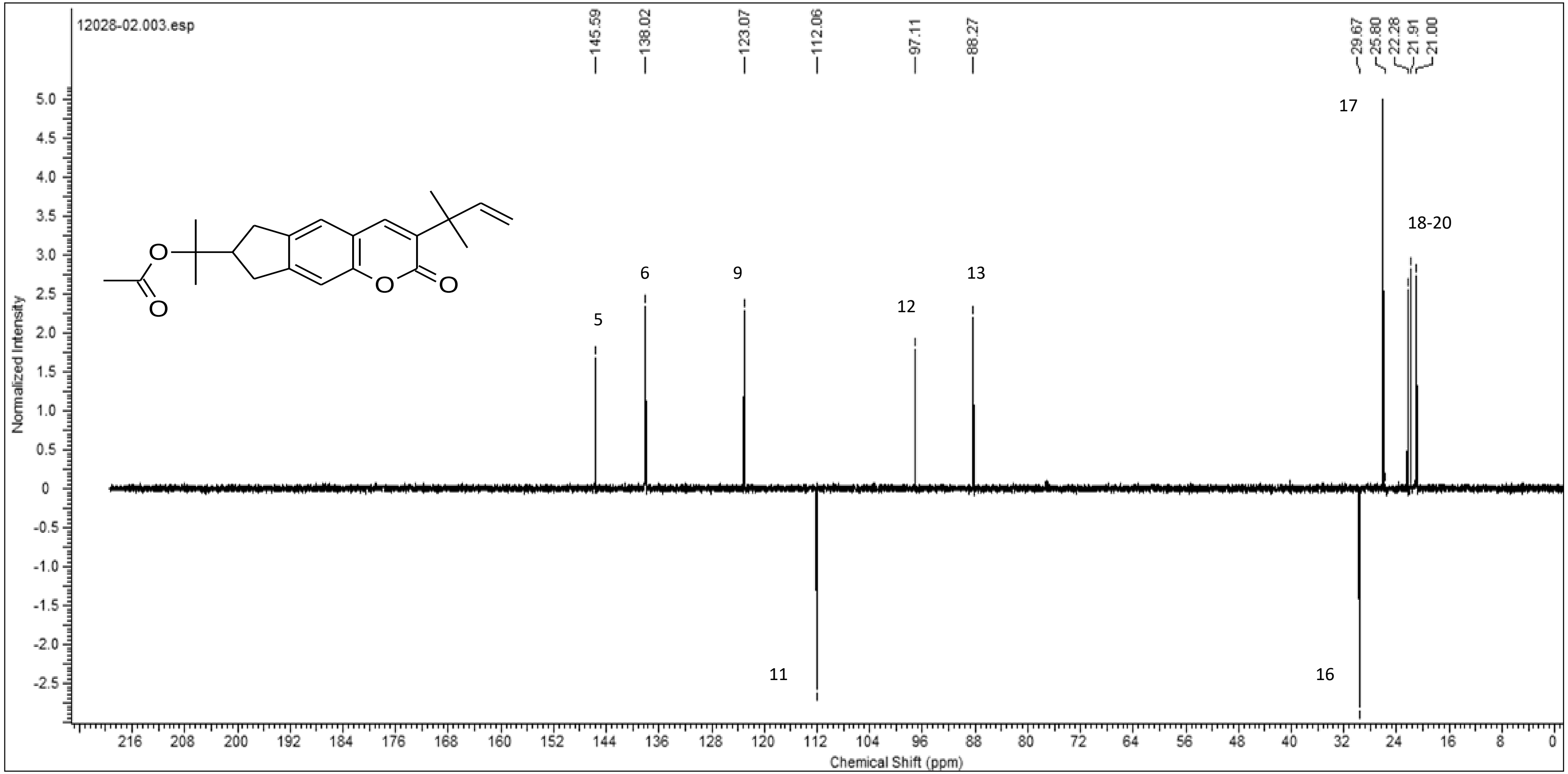

Figure S7. DEPT 135 spectrum from ^{13}C-NMR spectrum of **1**. Obtained by a Bruker Spectrometer Model Advance DPX400, 9.4 Teslas. Operating frequency 100 MHz. ($CDCl_3$).

Spectroscopic analysis of Rutamarin **1**

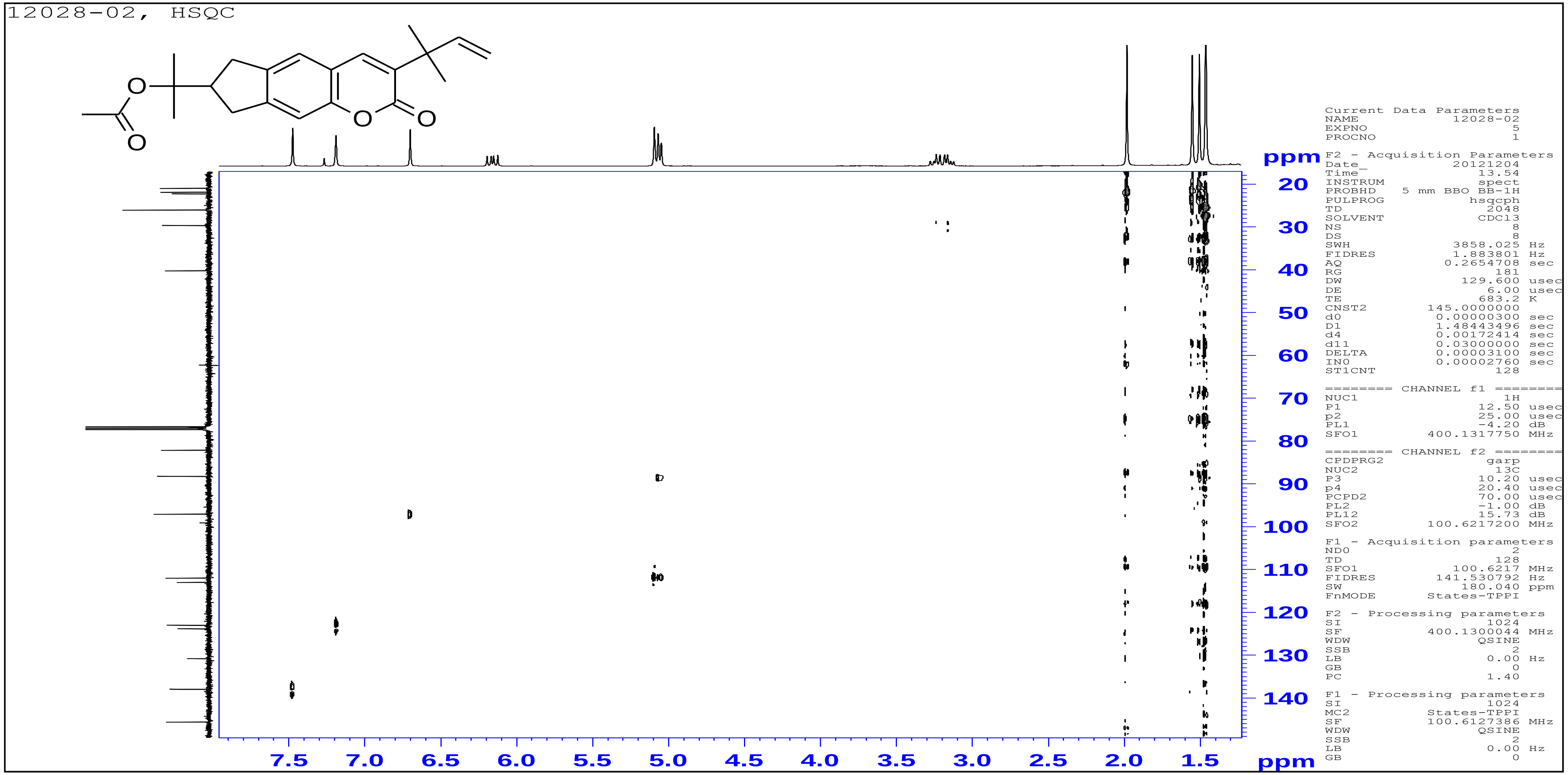

Figure S8. HSQC spectrum of **1**. Obtained by a Bruker Spectrometer Model Advance DPX400, 9.4 Teslas. Operating frequency 400 MHz.($CDCl_3$).

Spectroscopic analysis of Rutamarin **1**

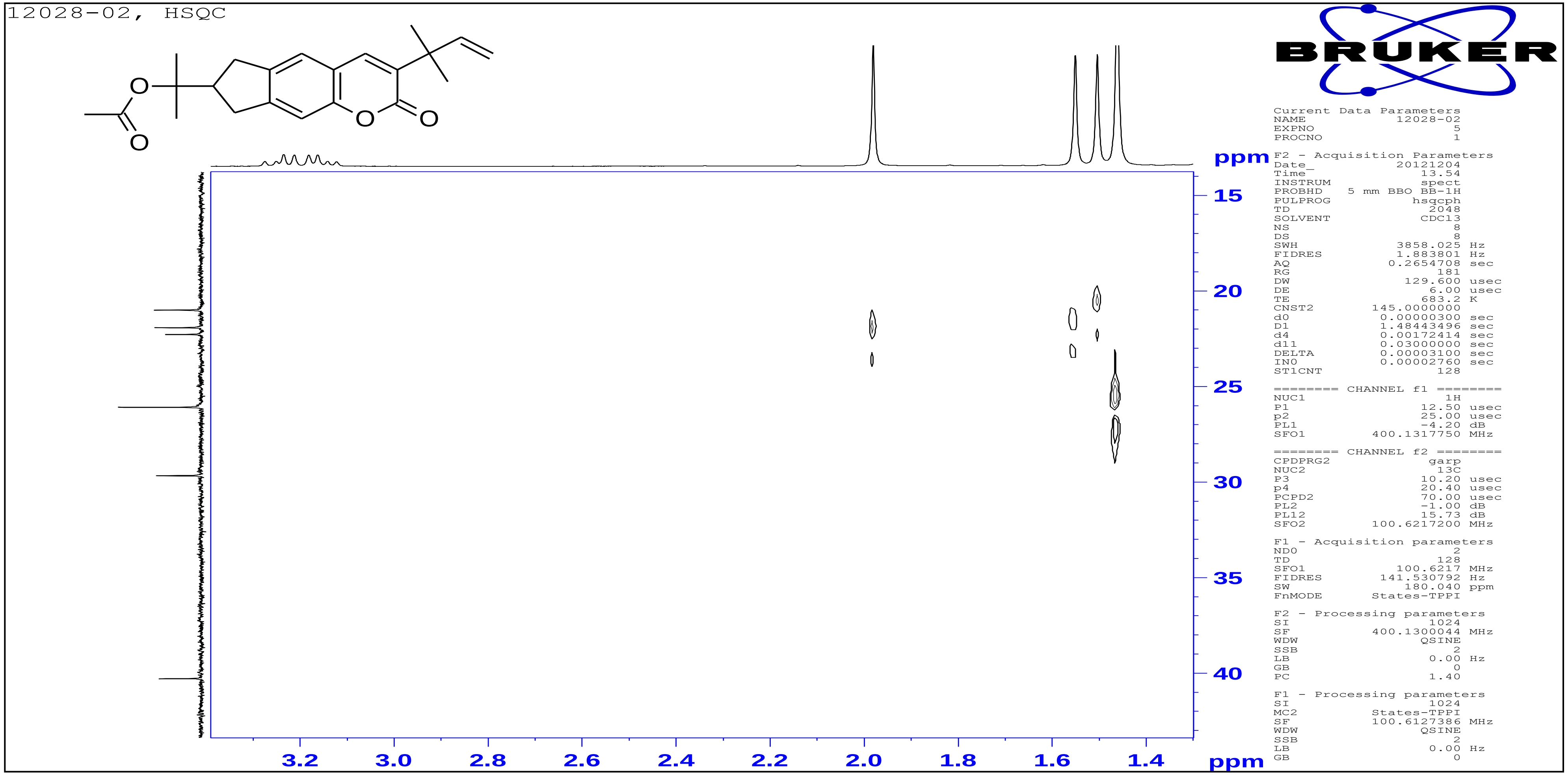

Figure S9. Expansion in the area ^{1}H: 1.3-3.4 ppm, ^{13}C: 15-45 ppm. HSQC spectrum of **1**. Obtained by a Bruker Spectrometer Model Advance DPX400, 9.4 Teslas. Operating frequency 400 MHz. ($CDCl_3$).

Spectroscopic analysis of Rutamarin **1**

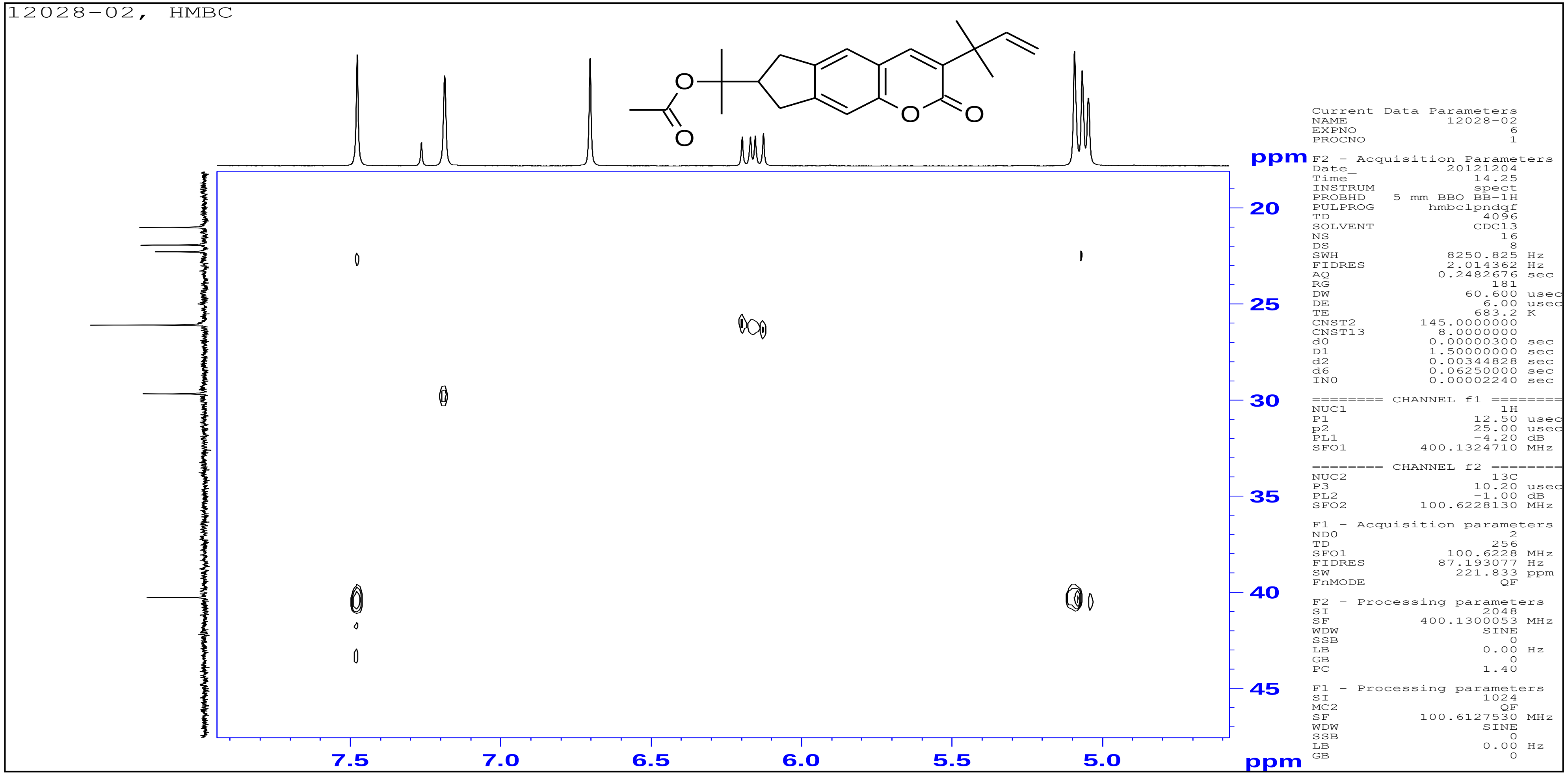

Figure S10. Expansion in the area ^{1}H: 4.6-8.0 ppm, ^{13}C: 20-50 ppm. HSQC spectrum of **1**. Obtained by a Bruker Spectrometer Model Advance DPX400, 9.4 Teslas. Operating frequency 400 MHz. ($CDCl_3$).

Spectroscopic analysis of Rutamarin **1**

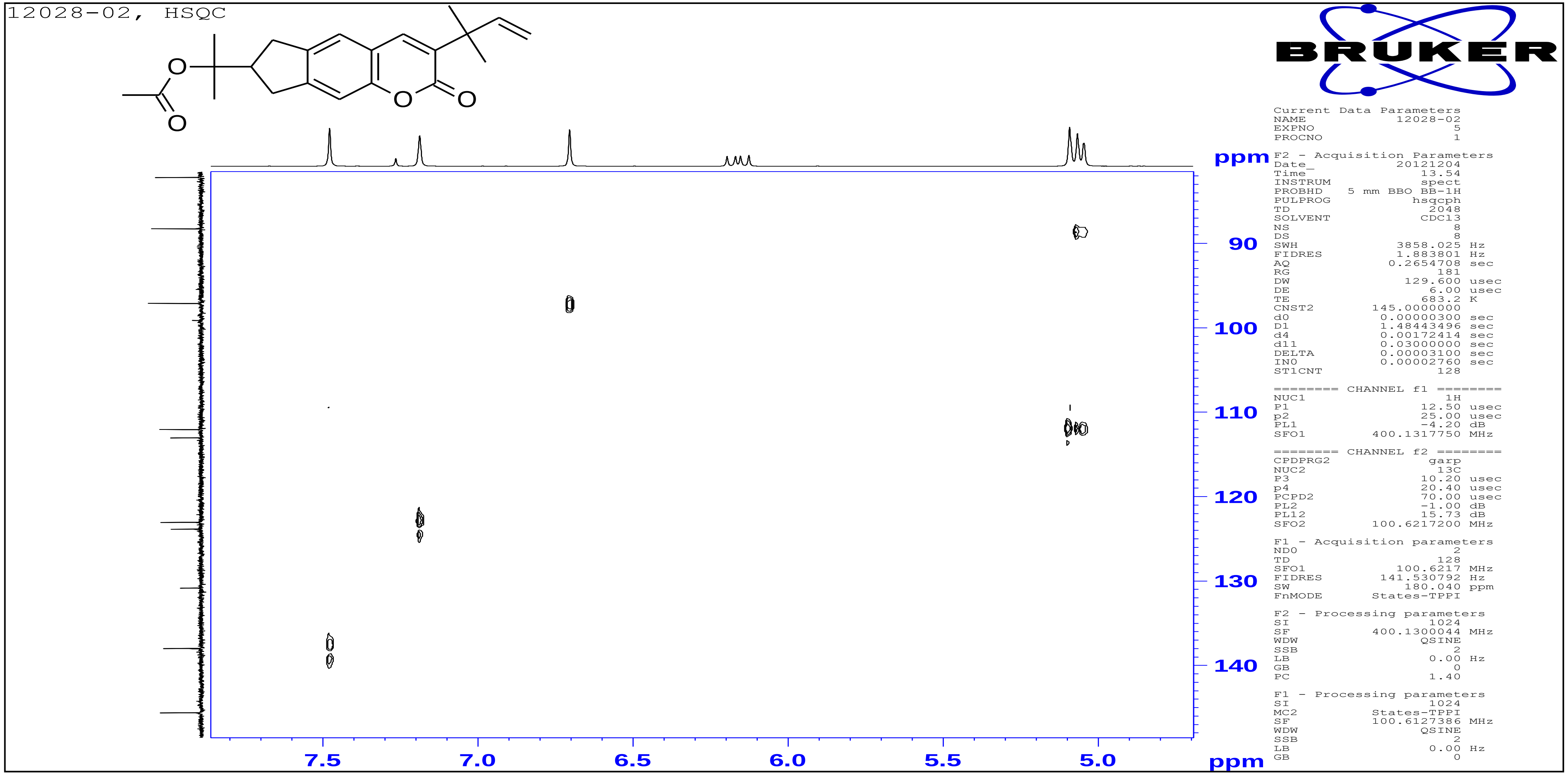

Figure S11. Expansion in the area ^{1}H: 4.6-8.0 ppm, ^{13}C: 80-150 ppm. HSQC spectrum of **1**. Obtained by a Bruker Spectrometer Model Advance DPX400, 9.4 Teslas. Operating frequency 400 MHz. ($CDCl_3$).

Spectroscopic analysis of Rutamarin **1**

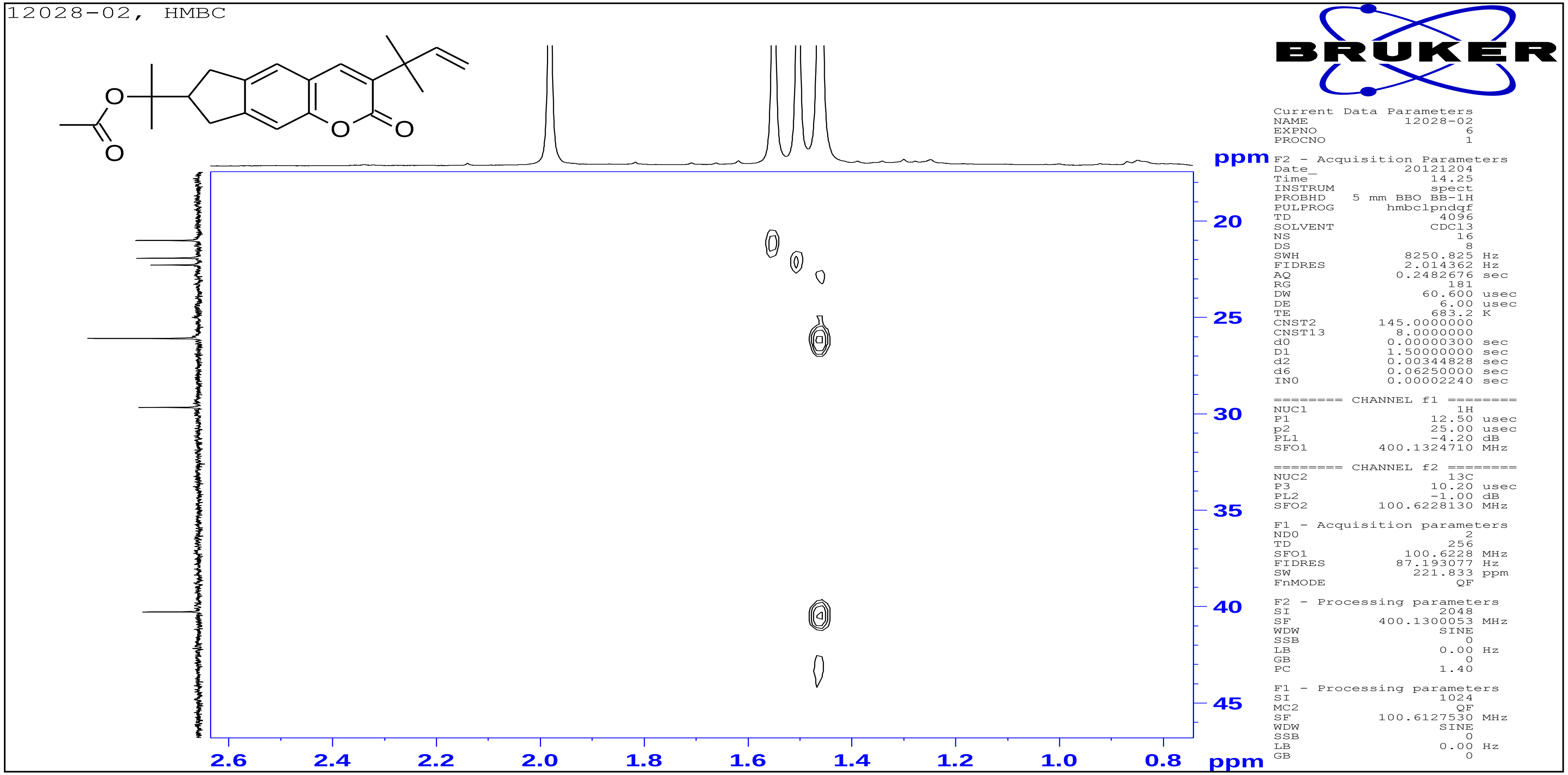

Figure S12. Expansion in the area ^{1}H: 0.8-2.6 ppm, ^{13}C: 15-50 ppm. HMBC spectrum of **1**. Obtained by a Bruker Spectrometer Model Advance DPX400, 9.4 Teslas. Operating frequency 400 MHz. ($CDCl_3$).

Spectroscopic analysis of Rutamarin **1**

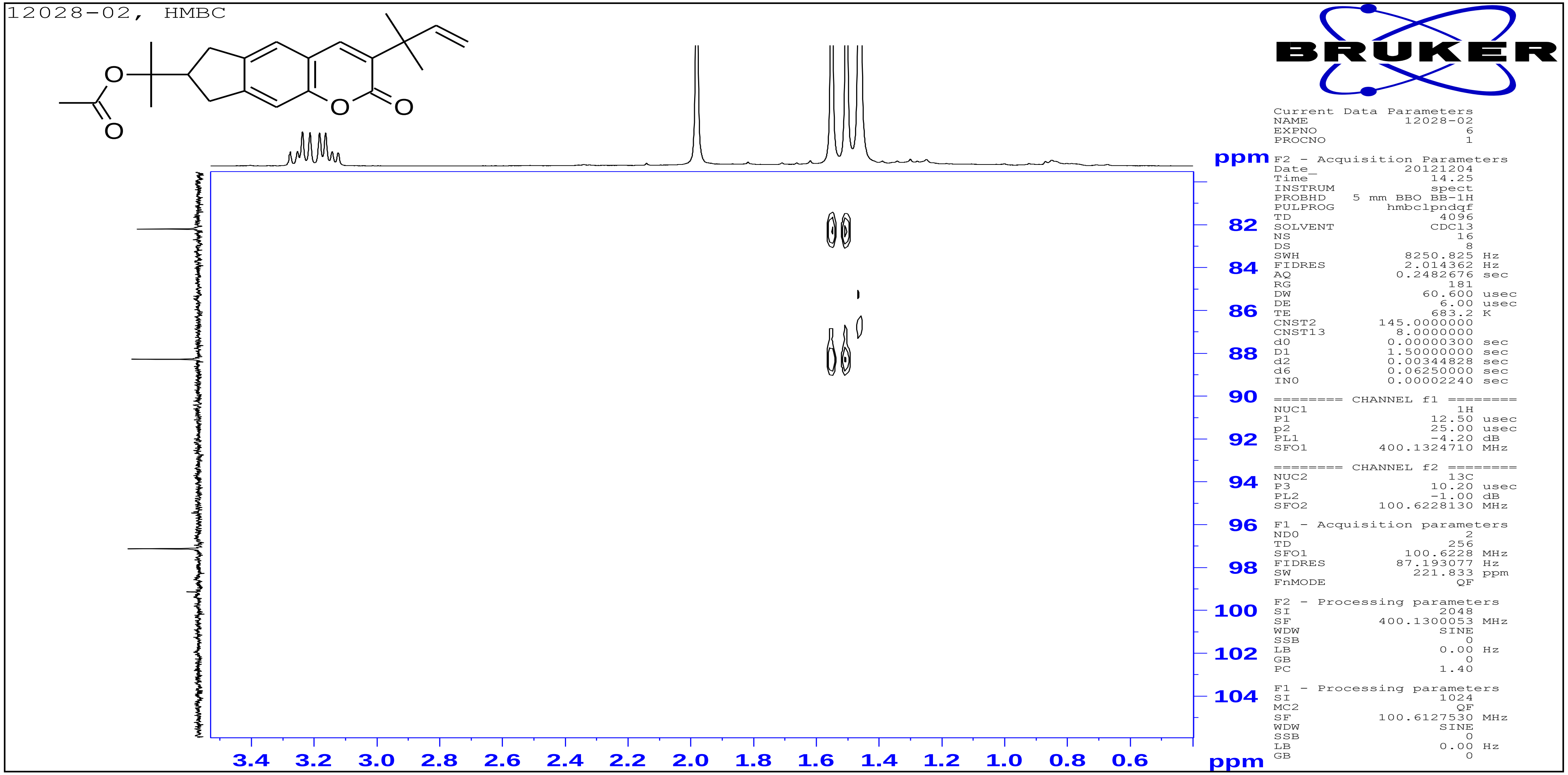

Figure S13. Expansion in the area ^{1}H: 0.4-3.5 ppm, ^{13}C: 80-106 ppm. HMBC spectrum of **1**. Obtained by a Bruker Spectrometer Model Advance DPX400, 9.4 Teslas. Operating frequency 400 MHz. ($CDCl_3$).

Spectroscopic analysis of Rutamarin **1**

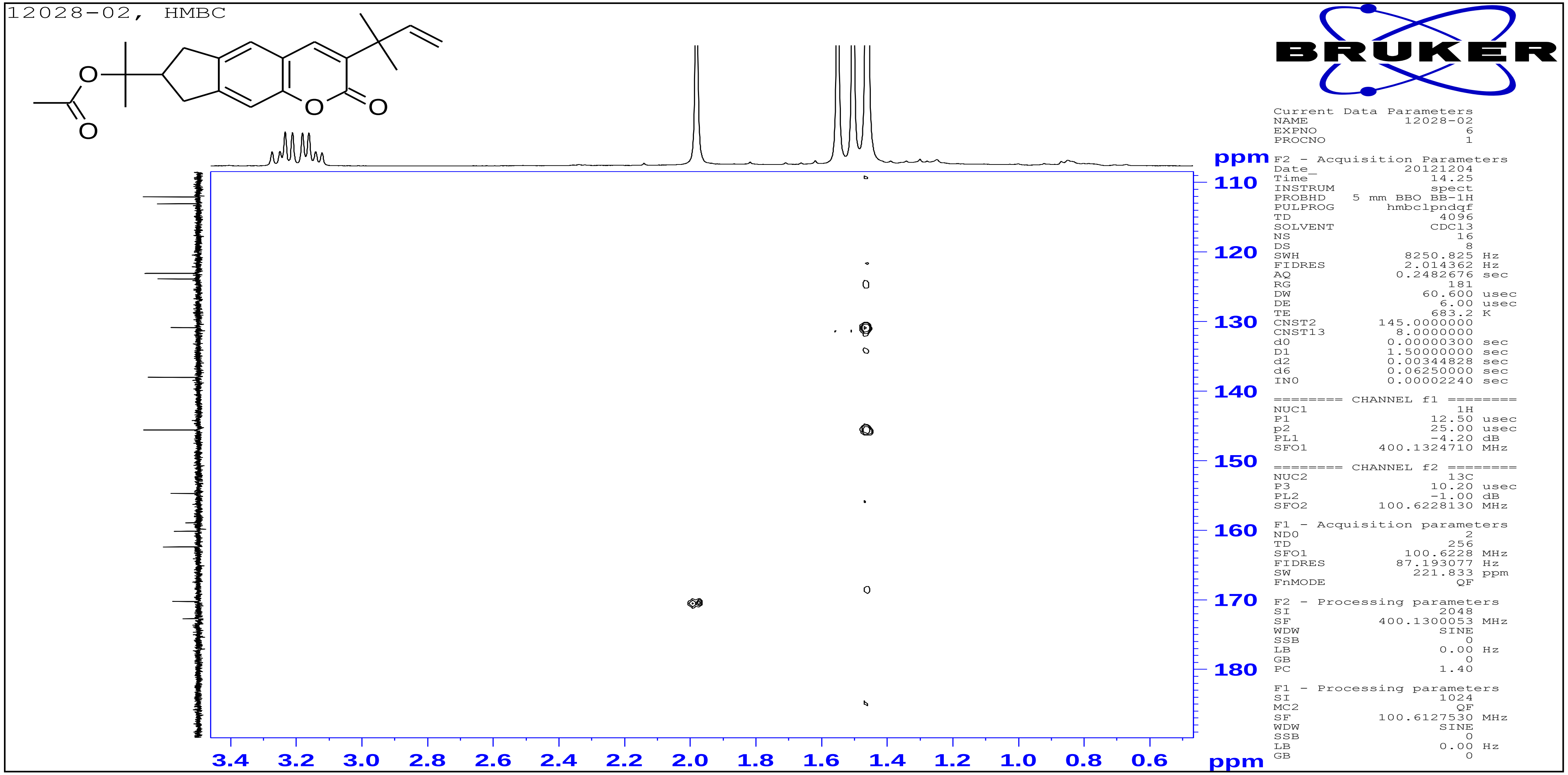

Figure S14. Expansion in the area ^{1}H: 0.4-3.4 ppm, ^{13}C: 110-190 ppm. HMBC spectrum of **1**. Obtained by a Bruker Spectrometer Model Advance DPX400, 9.4 Teslas. Operating frequency 400 MHz. ($CDCl_3$).

Spectroscopic analysis of Rutamarin **1**

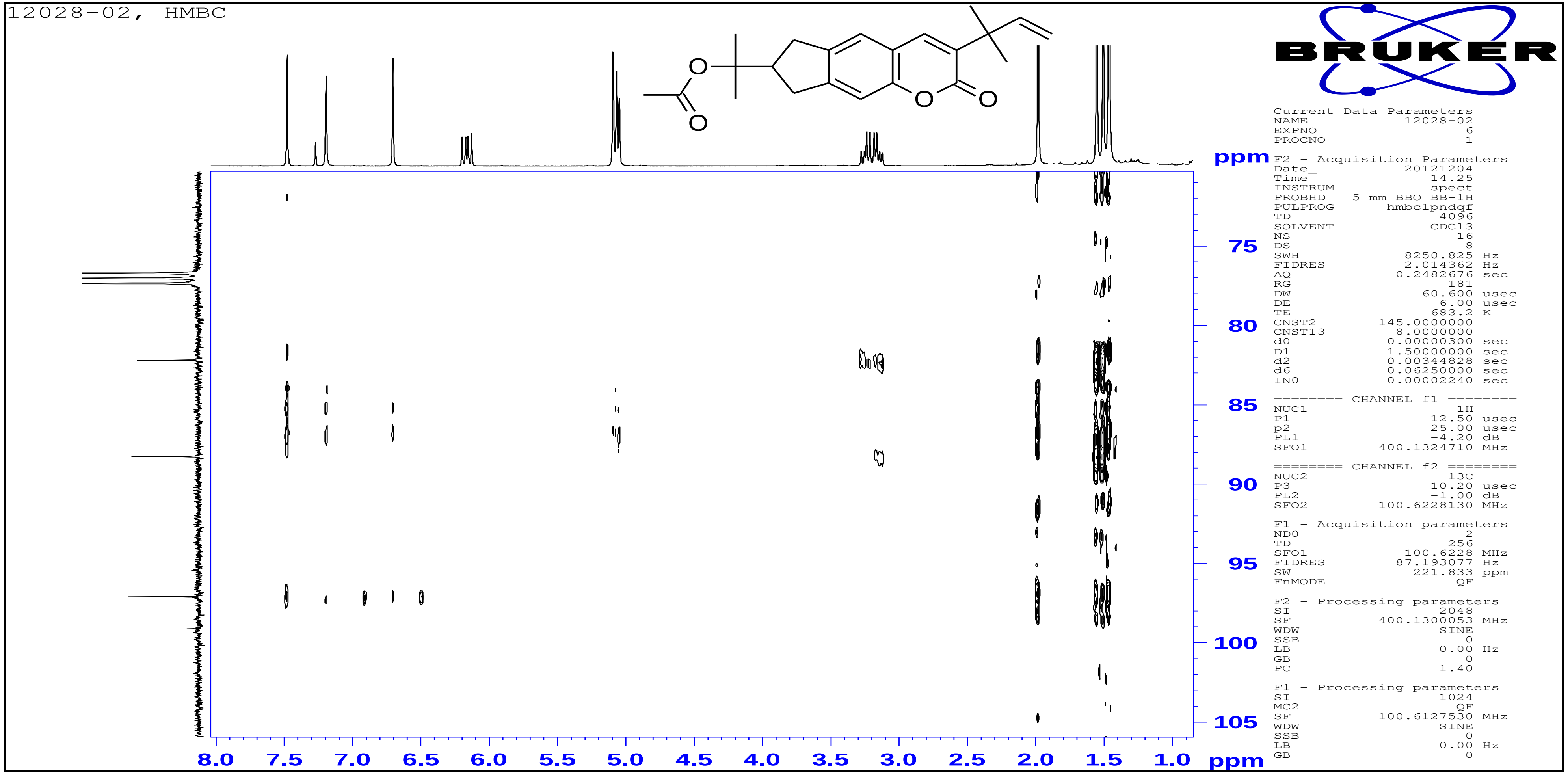

Figure S15. Expansion in the area ^{1}H: 1.8-8.0 ppm, ^{13}C: 70-105 ppm. HMBC spectrum of **1**. Obtained by a Bruker Spectrometer Model Advance DPX400, 9.4 Teslas. Operating frequency 400 MHz. ($CDCl_3$).

Spectroscopic analysis of Rutamarin **1**

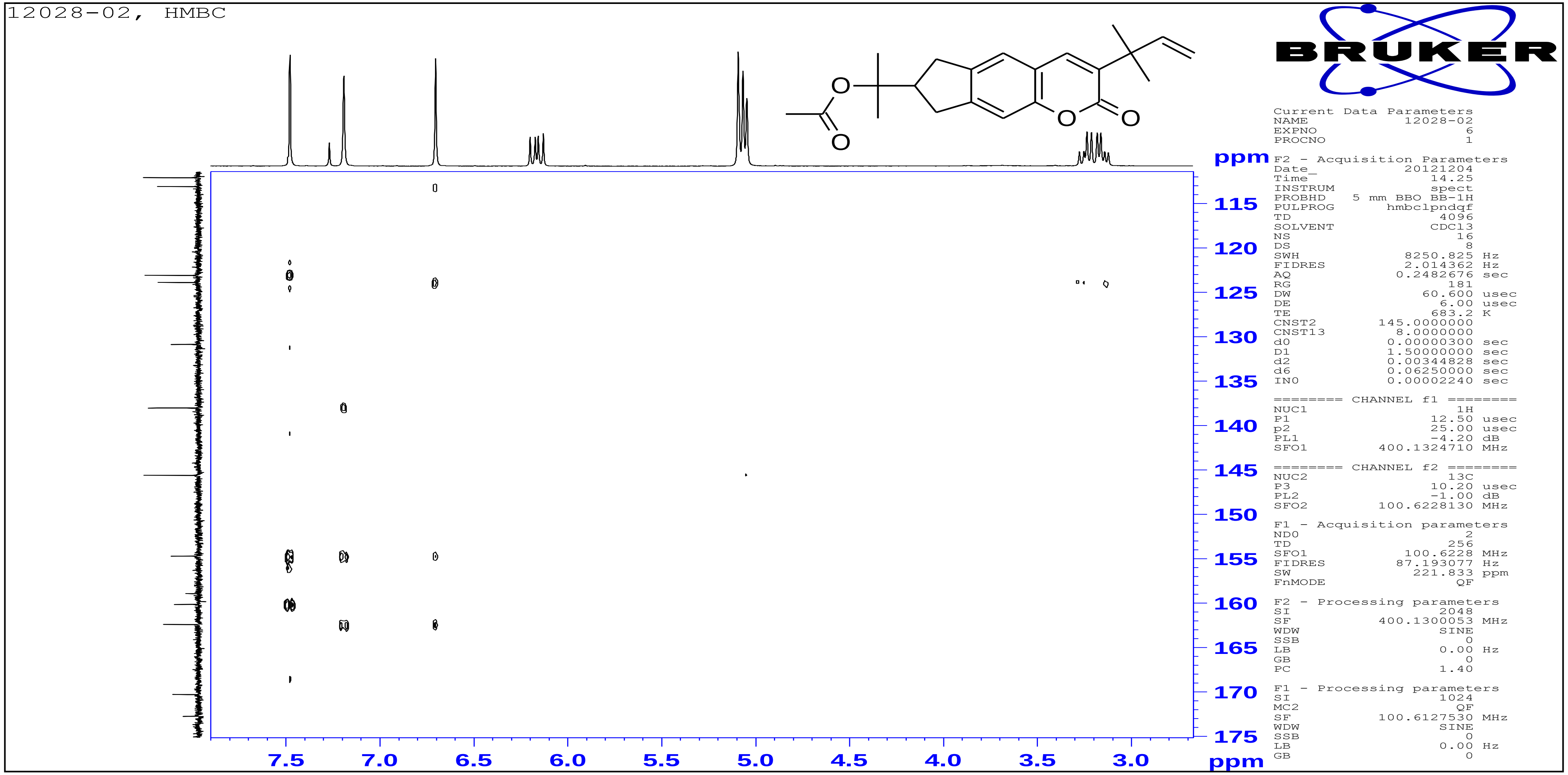

Figure S16. Expansion in the area ^{1}H: 2.5-8.0 ppm, ^{13}C: 110-175 ppm. HMBC spectrum of **1**. Obtained by a Bruker Spectrometer Model Advance DPX400, 9.4 Teslas. Operating frequency 400 MHz. ($CDCl_3$).

Spectroscopic analysis of Chalepin **2**

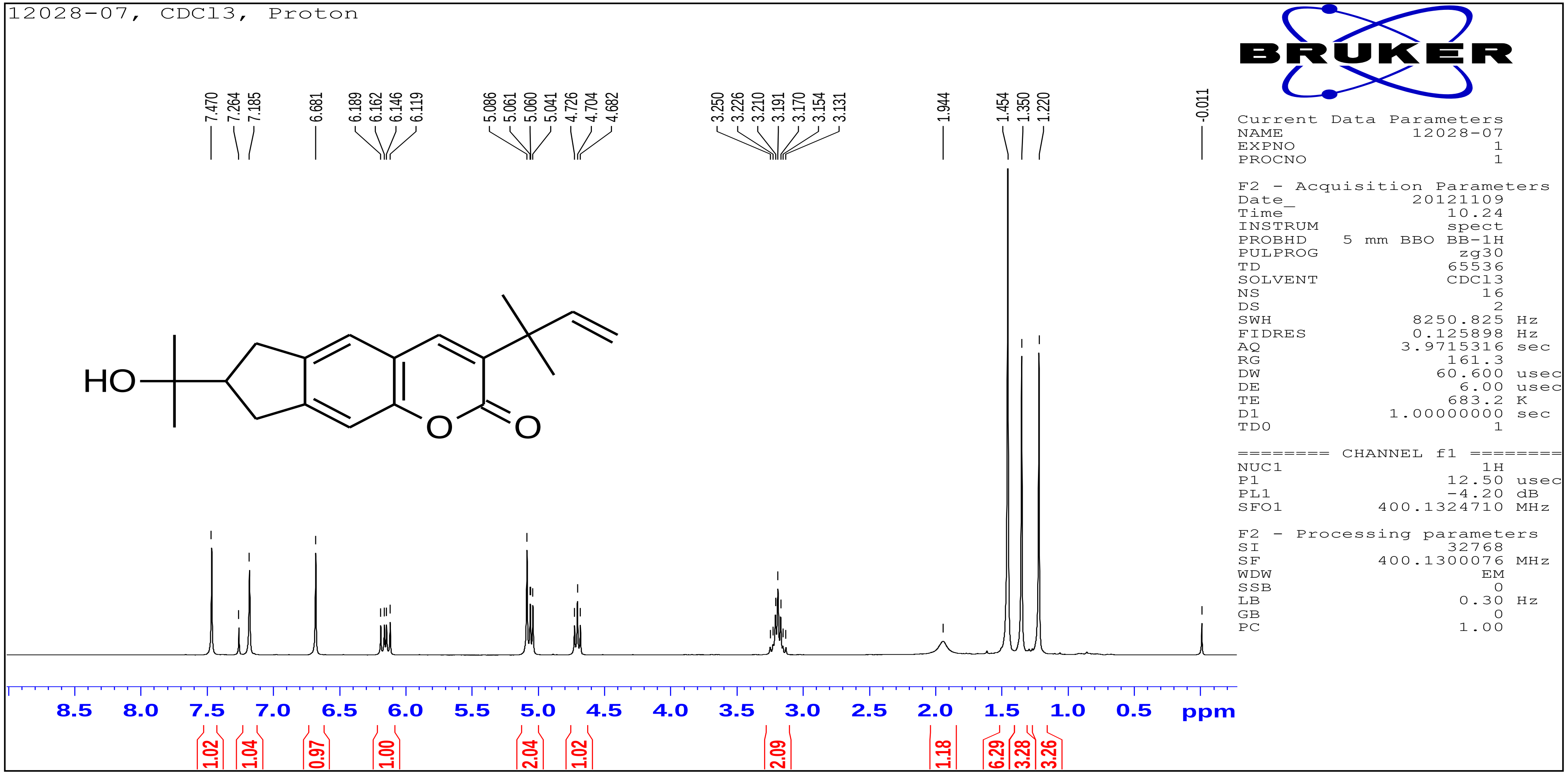

Figure S17. ^{1}H-NMR spectrum of **2**. Obtained by a Bruker Spectrometer Model Advance DPX400, 9.4 Teslas. Operating frequency 400 MHz. ($CDCl_3$).

Spectroscopic analysis of Chalepin **2**

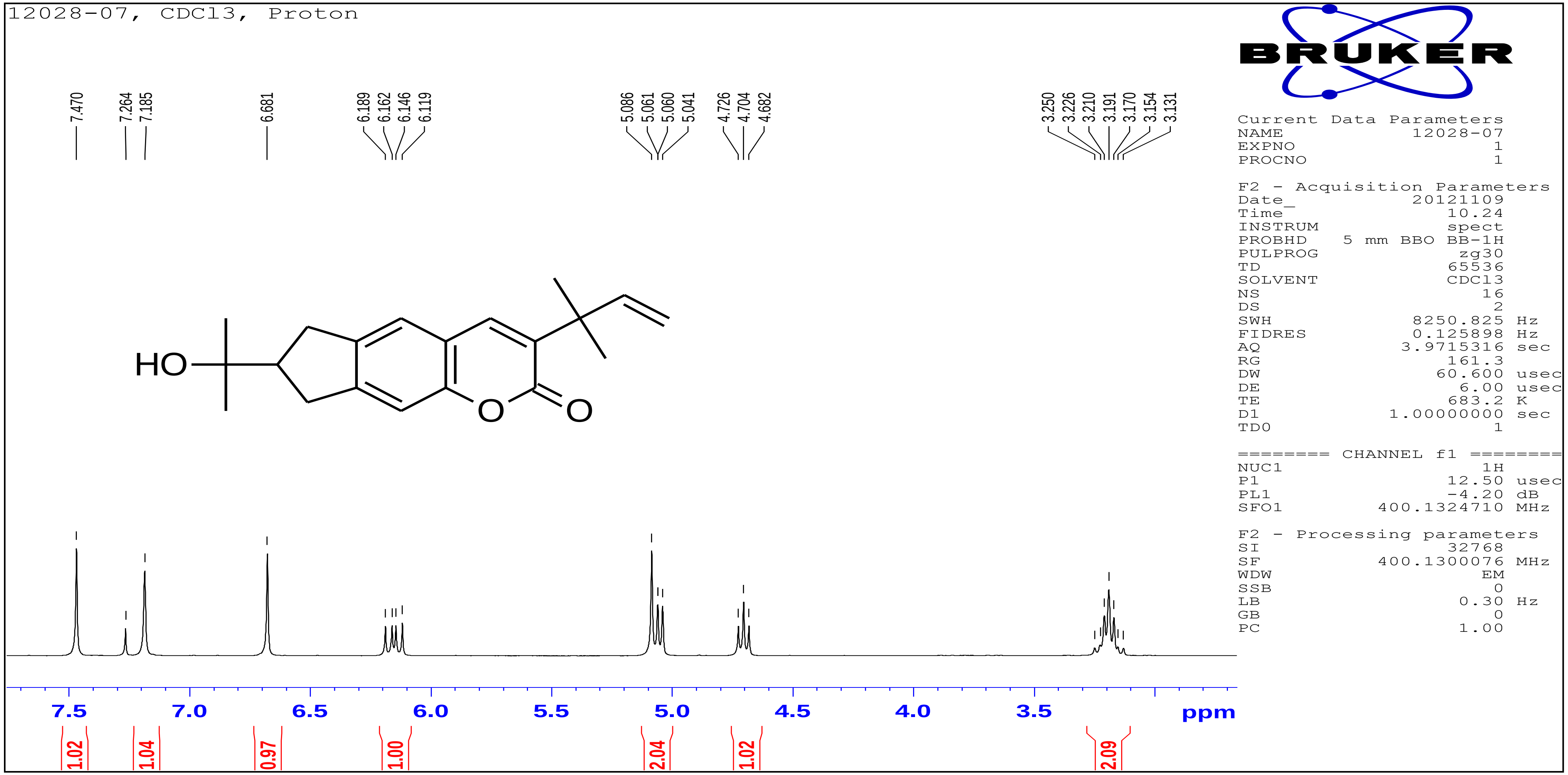

Figure S18. Expansion in the area from 2.5-8.0 ppm. ^{1}H-NMR spectrum of **2**. Obtained by a Bruker Spectrometer Model Advance DPX400, 9.4 Teslas. Operating frequency 400 MHz. ($CDCl_3$).

Spectroscopic analysis of Chalepin **2**

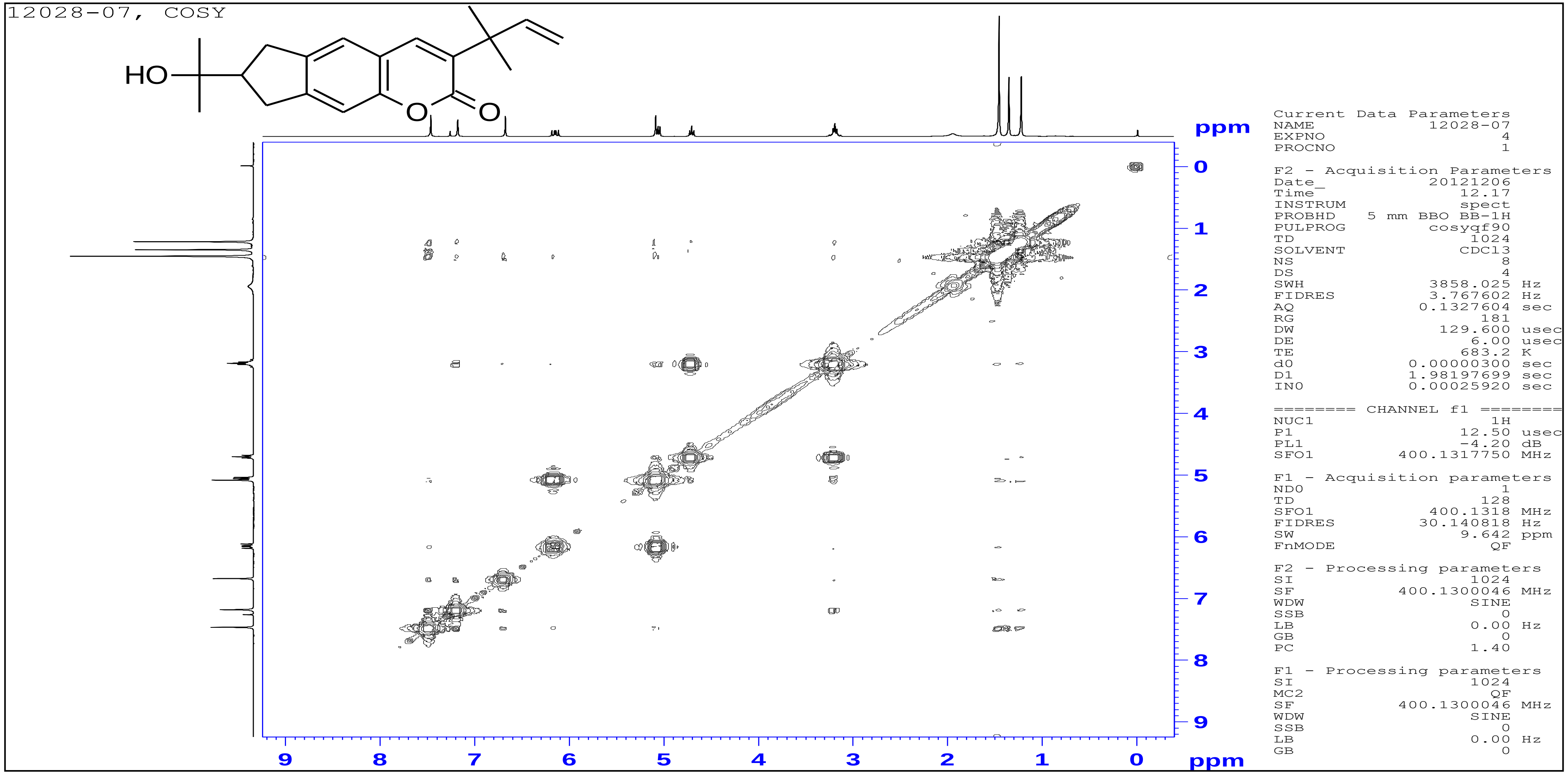

Figure S19. COSY spectrum of **2**. Obtained by a Bruker Spectrometer Model Advance DPX400, 9.4 Teslas. Operating frequency 400 MHz. ($CDCl_3$).

Spectroscopic analysis of Chalepin **2**

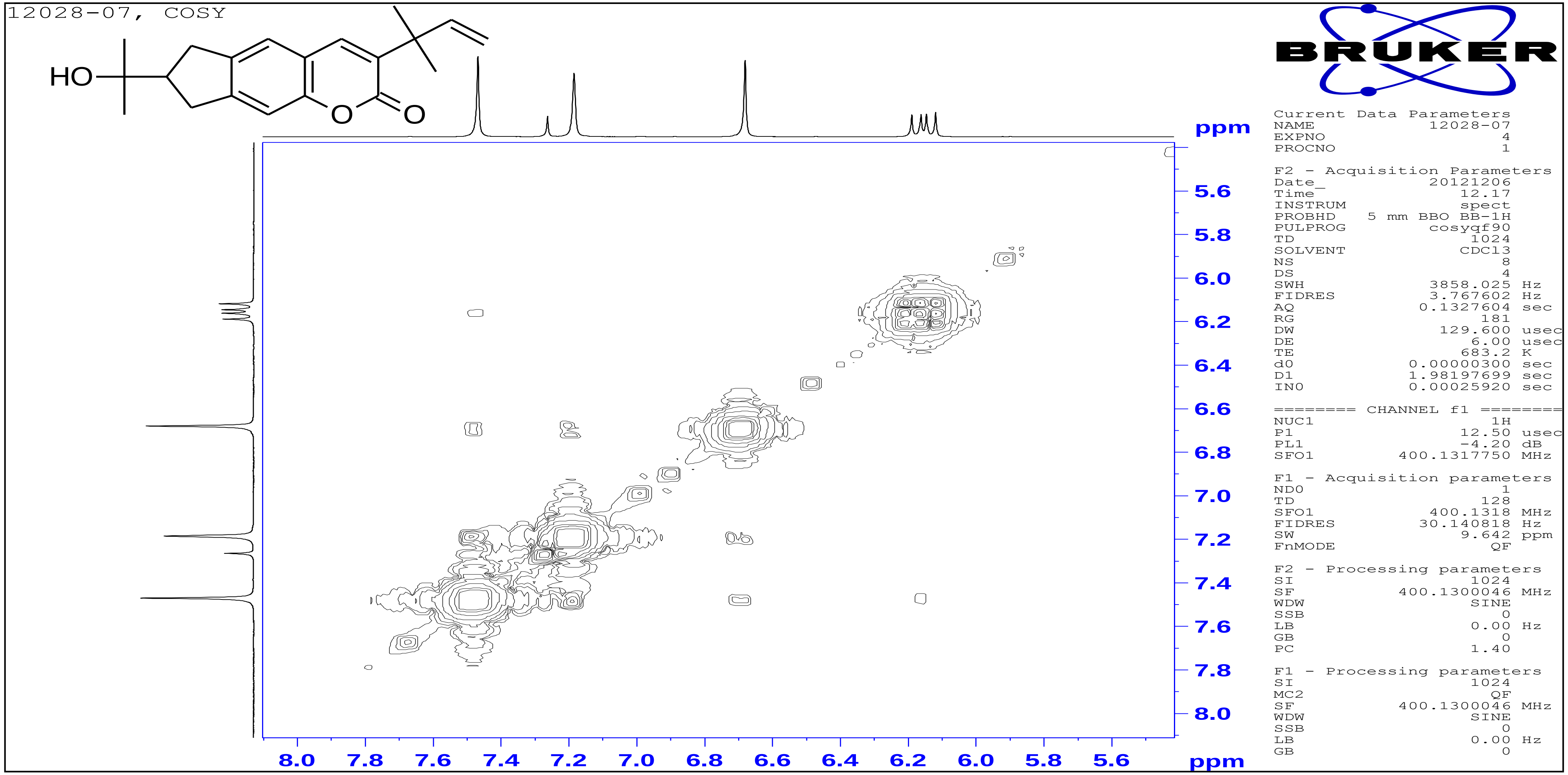

Figure S20. Expansion in the area ^{1}H: 5.6-8-0ppm, ^{1}H: 5.6-8.0 ppm. COSY spectrum of **2**. Obtained by a Bruker Spectrometer Model Advance DPX400, 9.4 Teslas. Operating frequency 400 MHz. ($CDCl_3$).

Spectroscopic analysis of Chalepin **2**

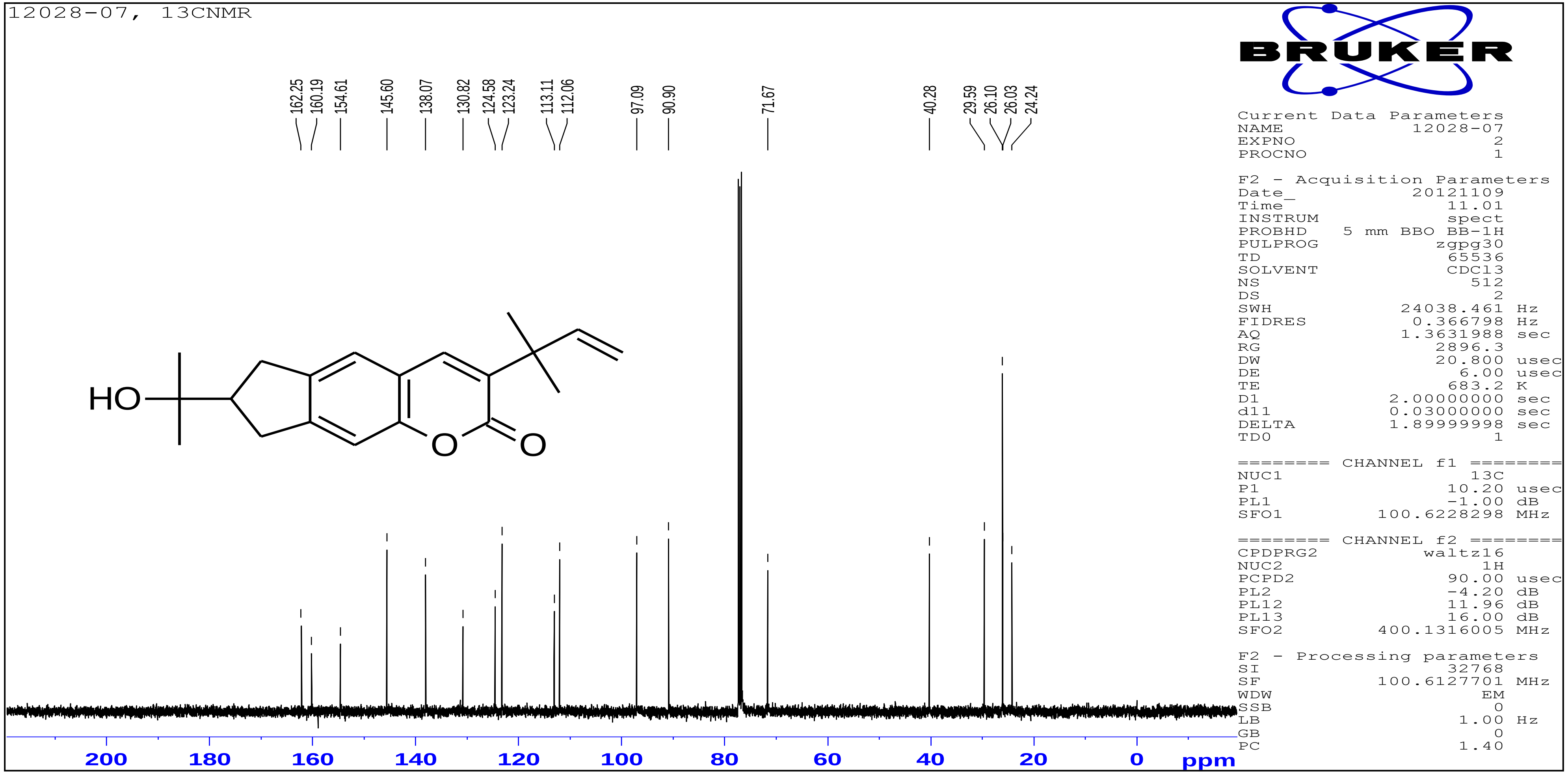

Figure S21. ^{13}C-NMR spectrum of **2**. Obtained by a Bruker Spectrometer Model Advance DPX400, 9.4 Teslas. Operating frequency 100 MHz. ($CDCl_3$).

Spectroscopic analysis of Chalepin **2**

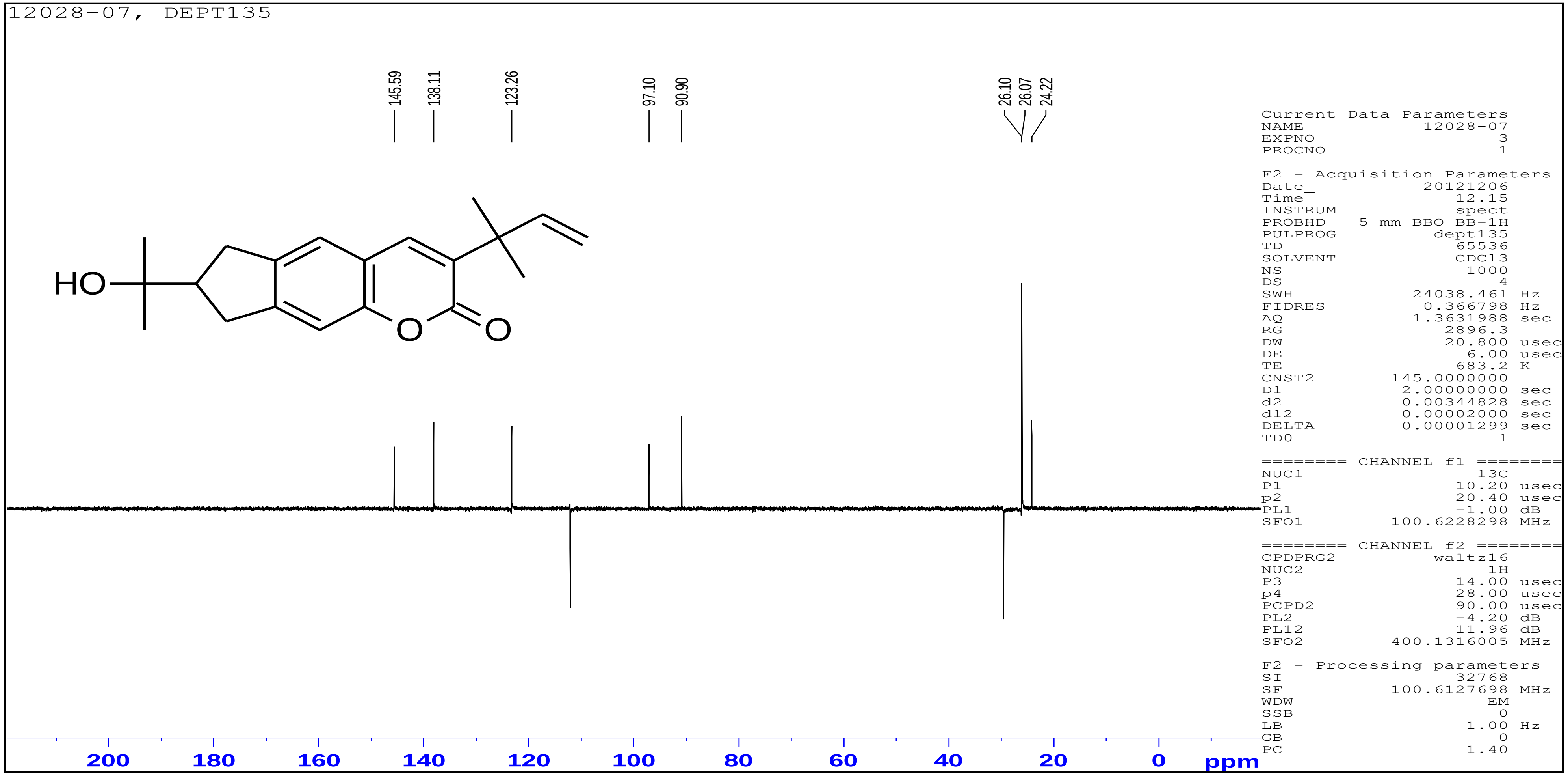

Figure S22. DEPT 135 spectrum from ^{13}C-NMR spectrum of **2**. Obtained by a Bruker Spectrometer Model Advance DPX400, 9.4 Teslas. Operating frequency 100 MHz. ($CDCl_3$).

Spectroscopic analysis of Chalepin **2**

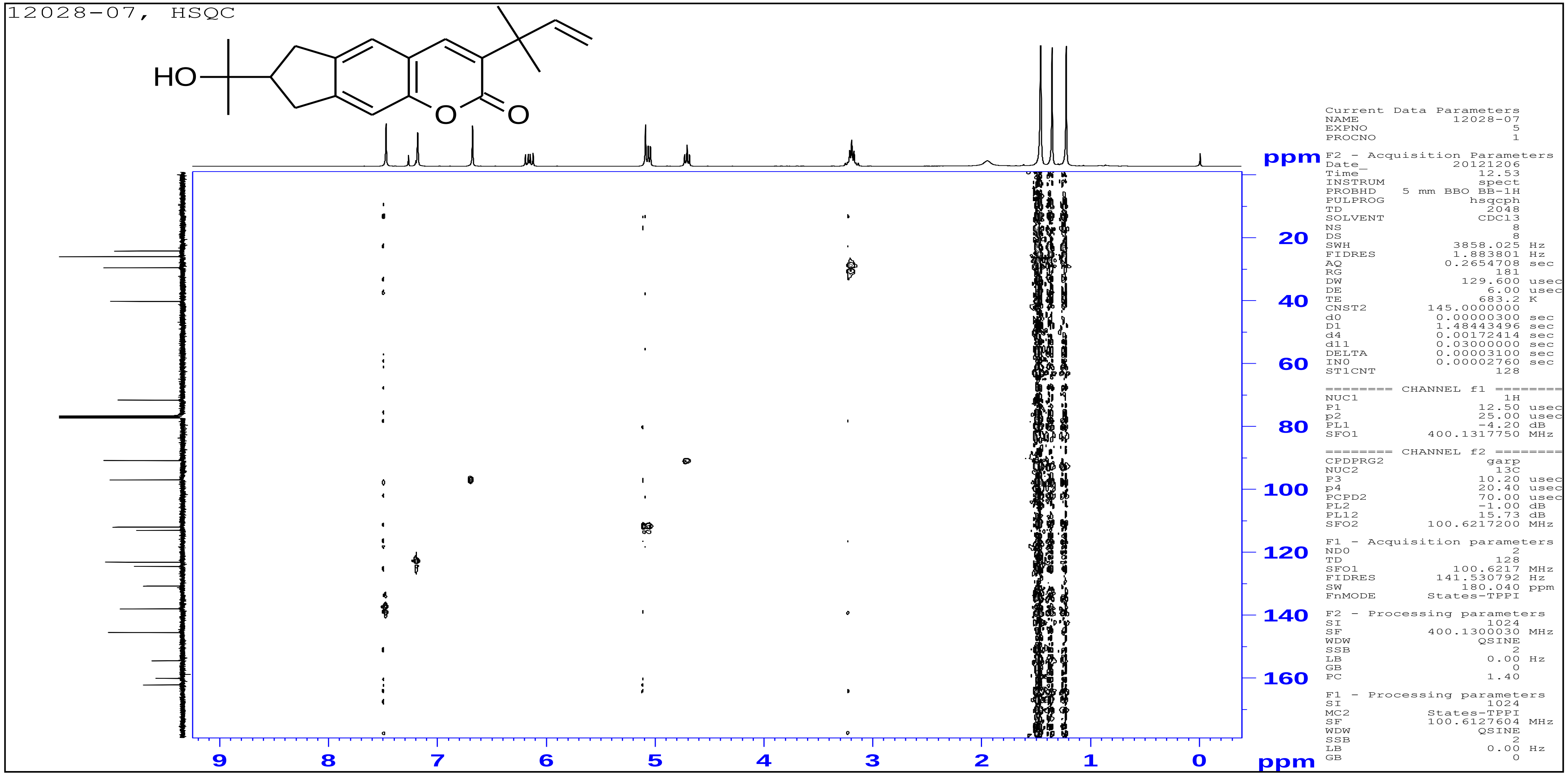

Figure S23. HSQC spectrum of **2**. Obtained by a Bruker Spectrometer Model Advance DPX400, 9.4 Teslas. Operating frequency 400 MHz. ($CDCl_3$).

Spectroscopic analysis of Chalepin **2**

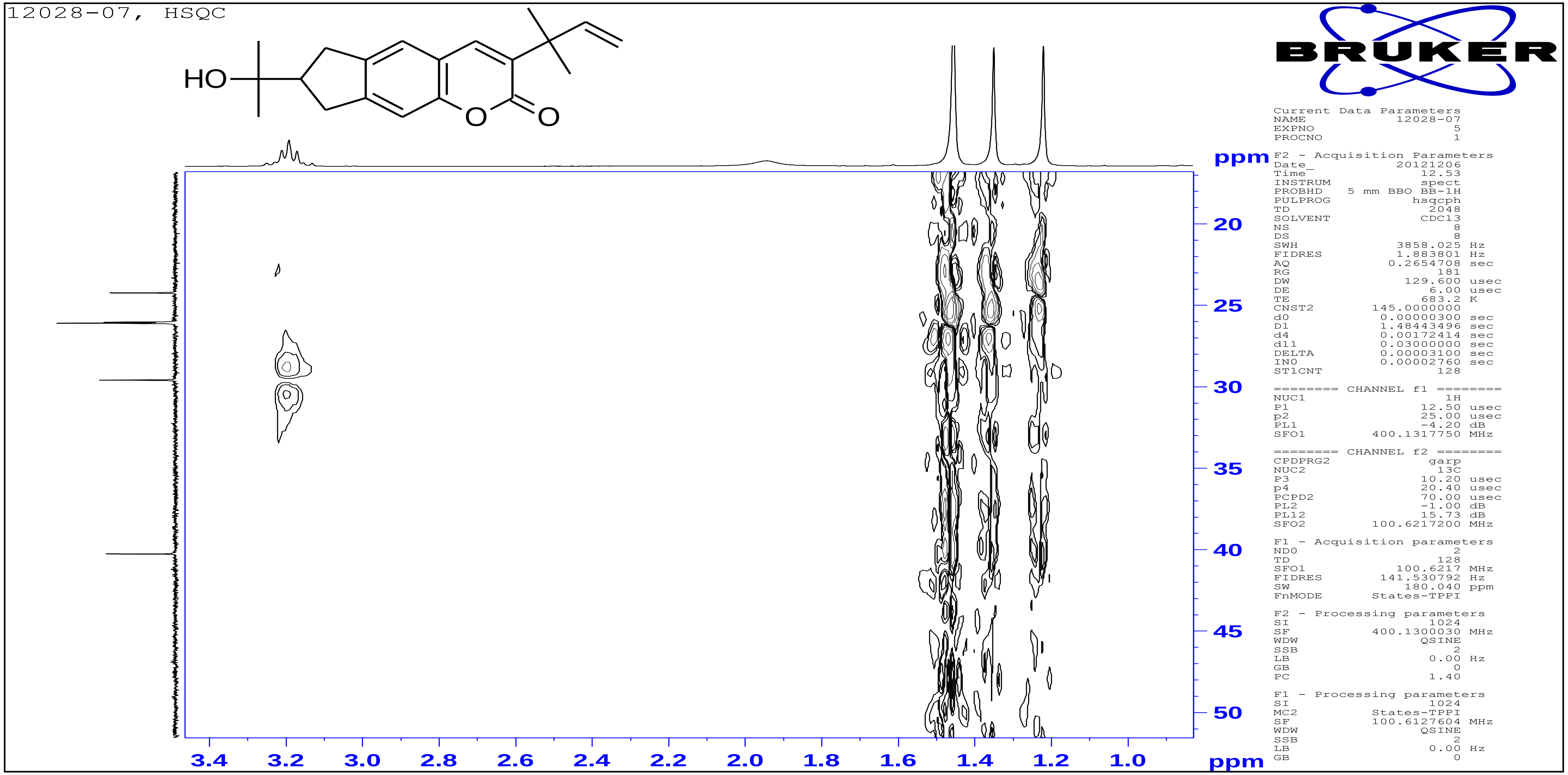

Figure S24. Expansion in the area ^{1}H: 1.0-3.4 ppm, ^{13}C: 15-50 ppm. HSQC spectrum of **2**. Obtained by a Bruker Spectrometer Model Advance DPX400, 9.4 Teslas. Operating frequency 400 MHz. ($CDCl_3$).

Spectroscopic analysis of Chalepin **2**

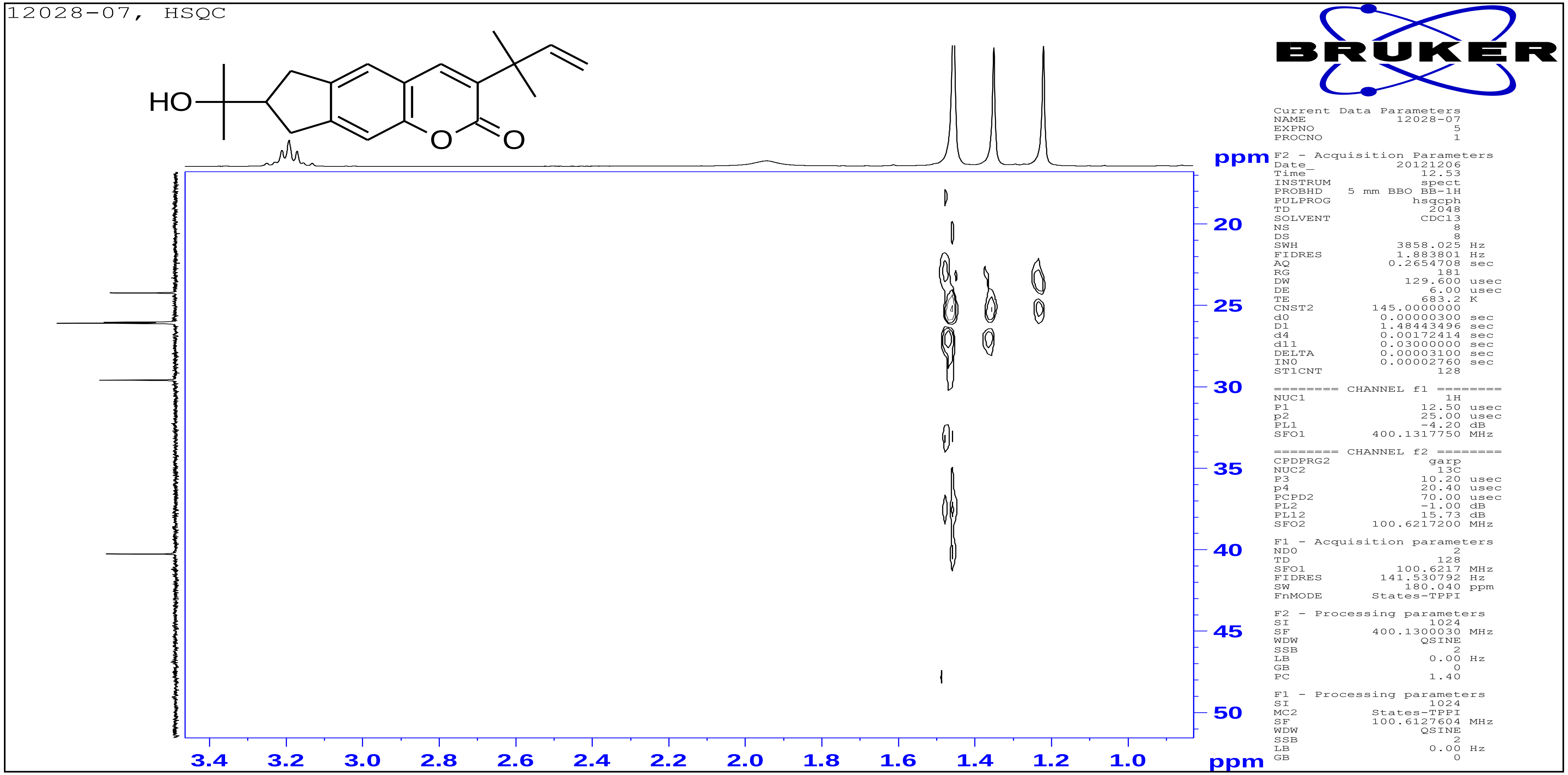

Figure S25. Expansion in the area ^{1}H: 1.0-3.4 ppm, ^{13}C: 15-50 ppm. HSQC spectrum of **2**. Obtained by a Bruker Spectrometer Model Advance DPX400, 9.4 Teslas. Operating frequency 400 MHz. ($CDCl_3$).

Spectroscopic analysis of Chalepin **2**

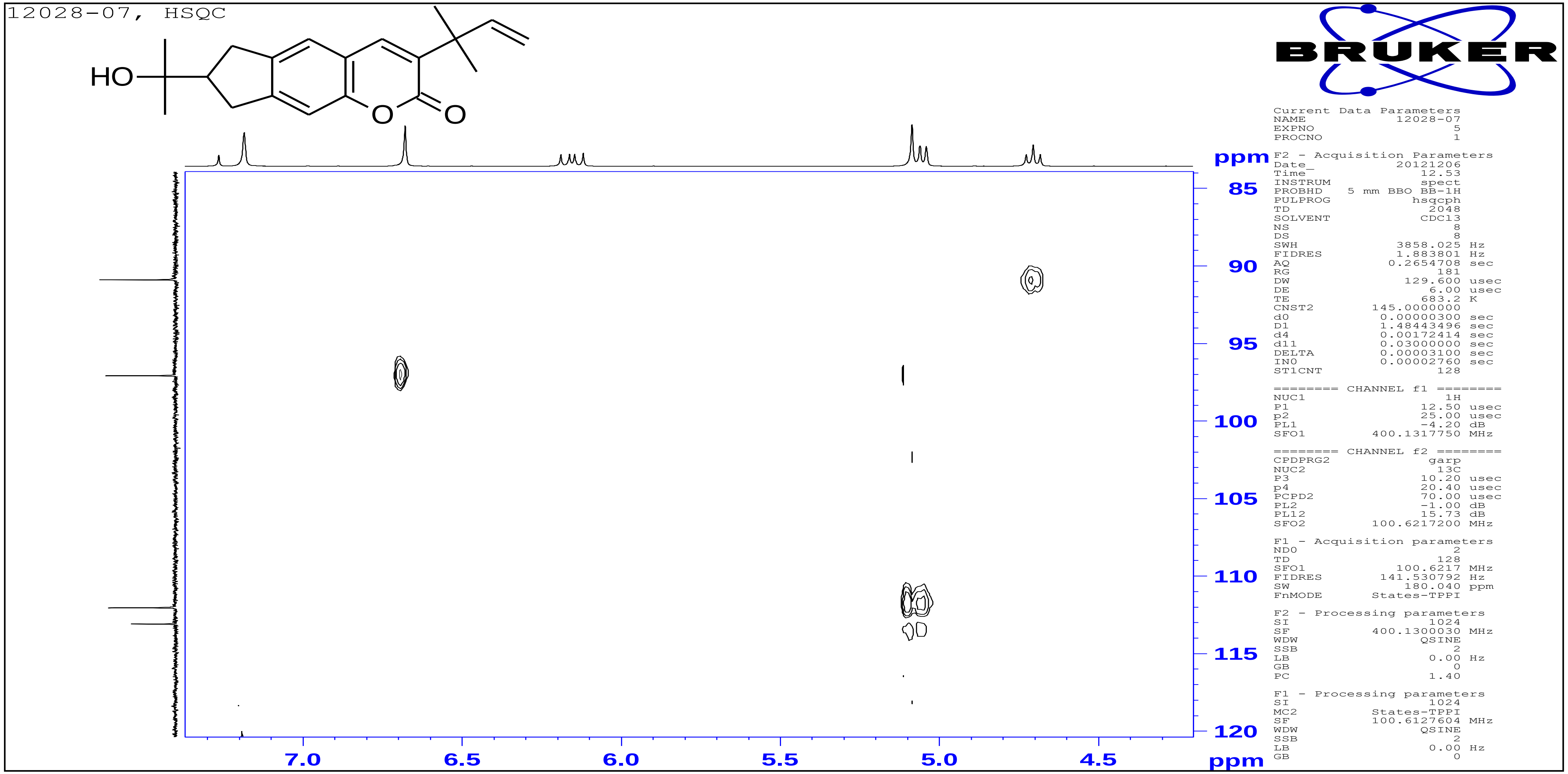

Figure S26. Expansion in the area ^{1}H: 4.0-7.5 ppm, ^{13}C: 85-120 ppm. HSQC spectrum of **2**. Obtained by a Bruker Spectrometer Model Advance DPX400, 9.4 Teslas. Operating frequency 400 MHz. ($CDCl_3$).

Spectroscopic analysis of Chalepin **2**

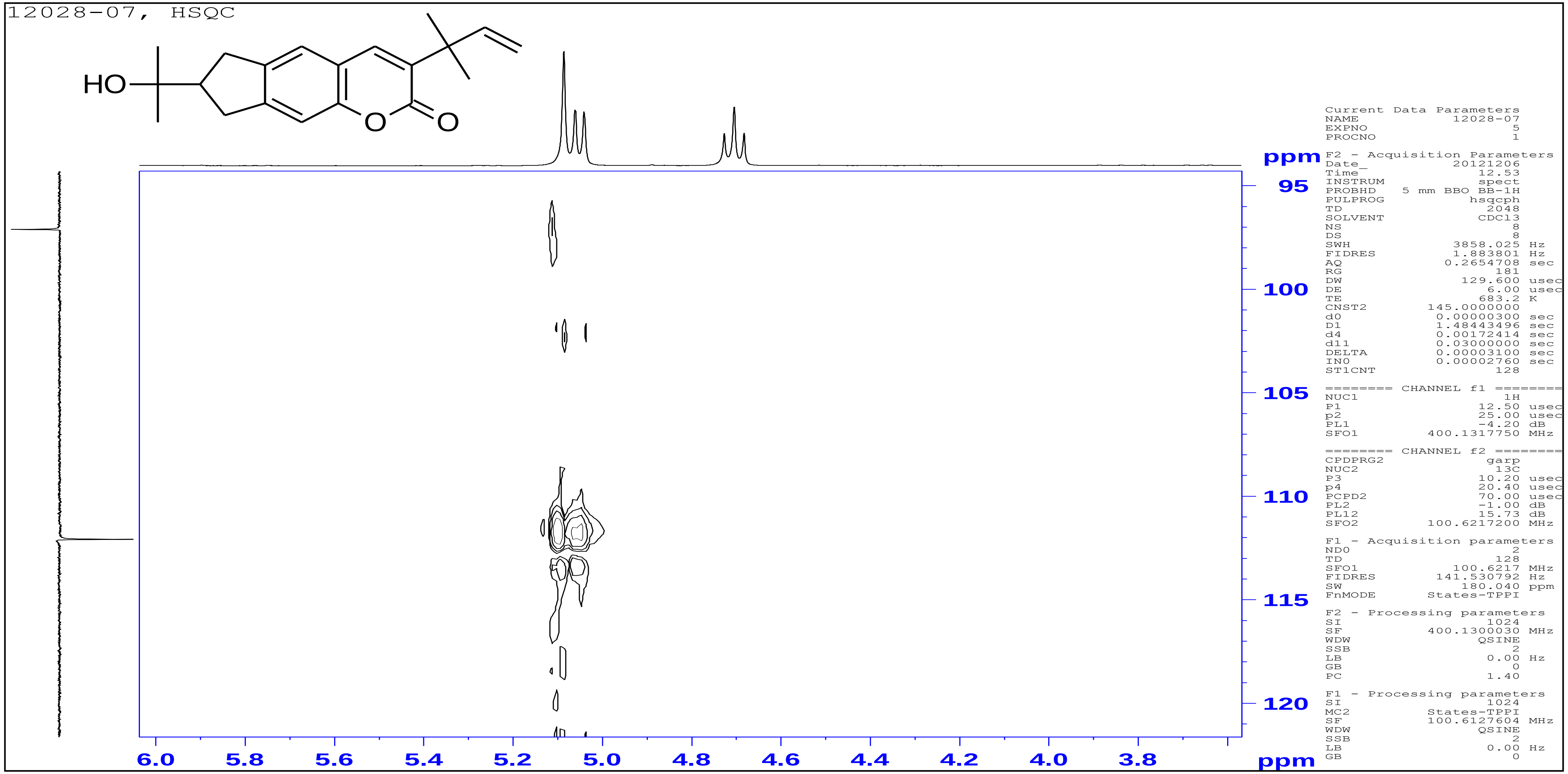

Figure S27. Expansion in the area ^{1}H: 3.6-6.0 ppm, ^{13}C: 95-120 ppm. HSQC spectrum of **2**. Obtained by a Bruker Spectrometer Model Advance DPX400, 9.4 Teslas. Operating frequency 400 MHz. ($CDCl_3$).

Spectroscopic analysis of Chalepin **2**

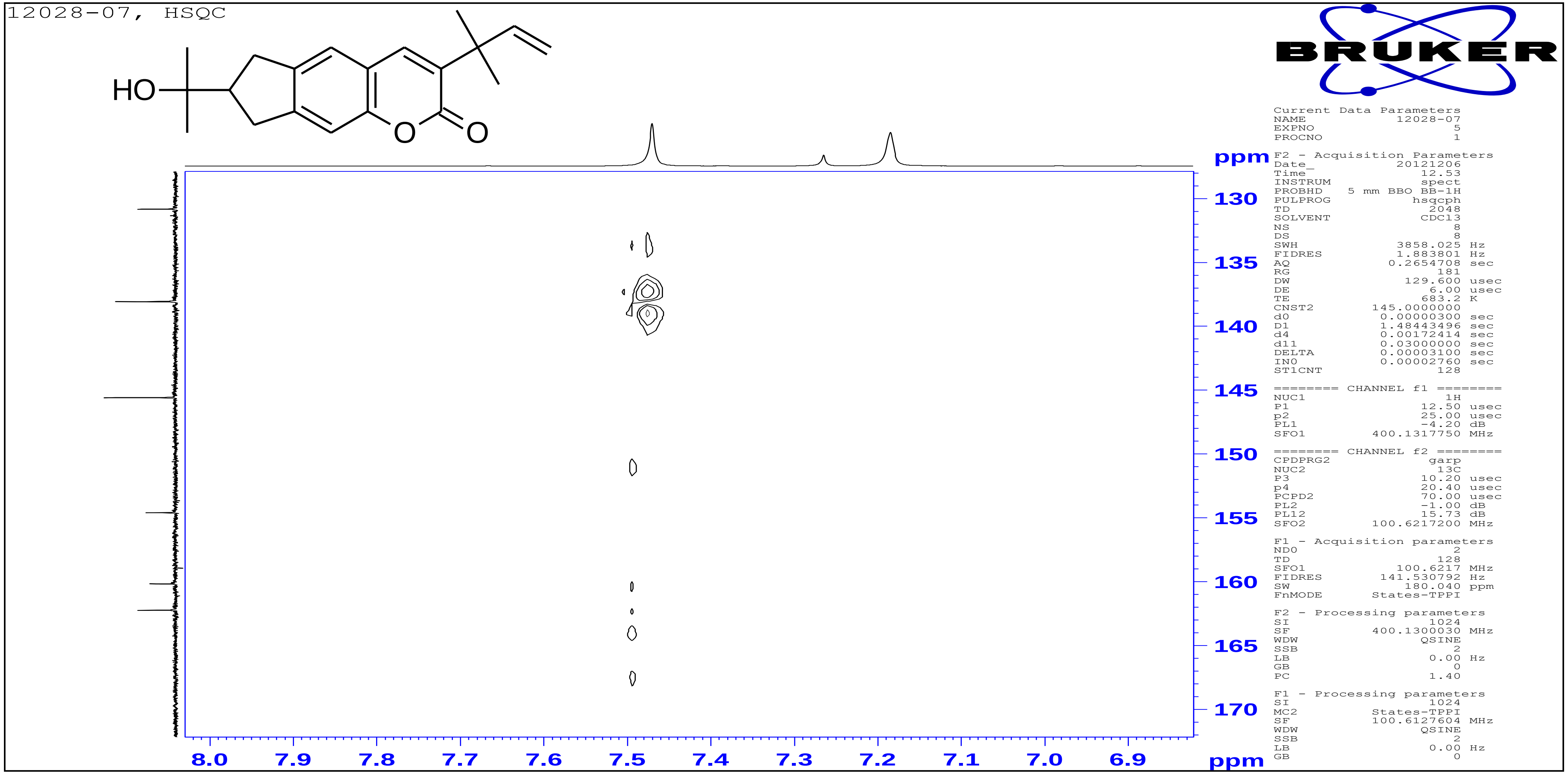

Figure S28. Expansion in the area ^{1}H: 6.8-8.0 ppm, ^{13}C: 130-170 ppm. HSQC spectrum of **2**. Obtained by a Bruker Spectrometer Model Advance DPX400, 9.4 Teslas. Operating frequency 400 MHz. ($CDCl_3$).

Spectroscopic analysis of Chalepin **2**

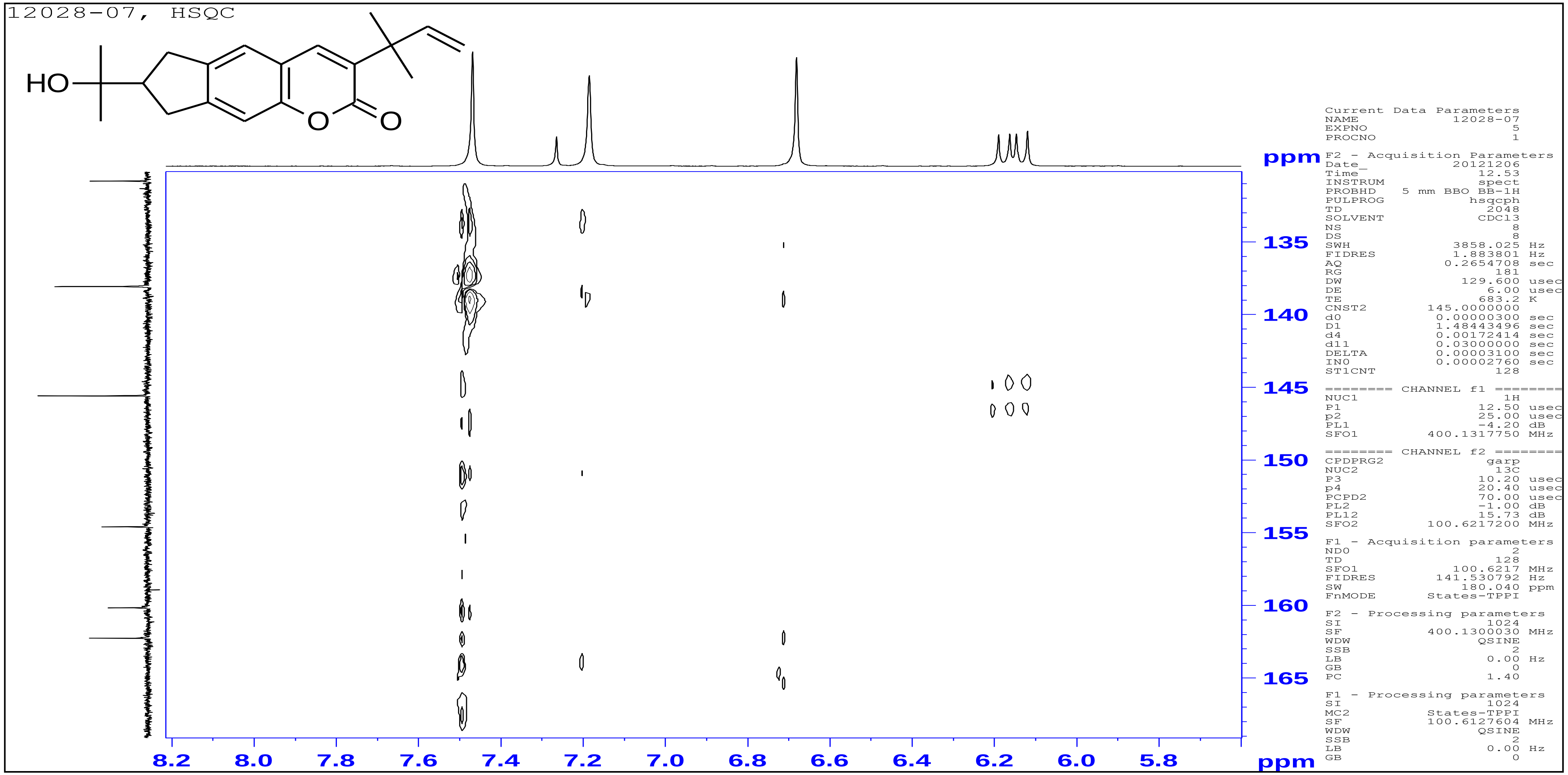

Figure S29. Expansion in the area ^{1}H: 5.6-8.2 ppm, ^{13}C: 130-170 ppm. HSQC spectrum of **2**. Obtained by a Bruker Spectrometer Model Advance DPX400, 9.4 Teslas. Operating frequency 400 MHz. ($CDCl_3$).

Spectroscopic analysis of Chalepin **2**

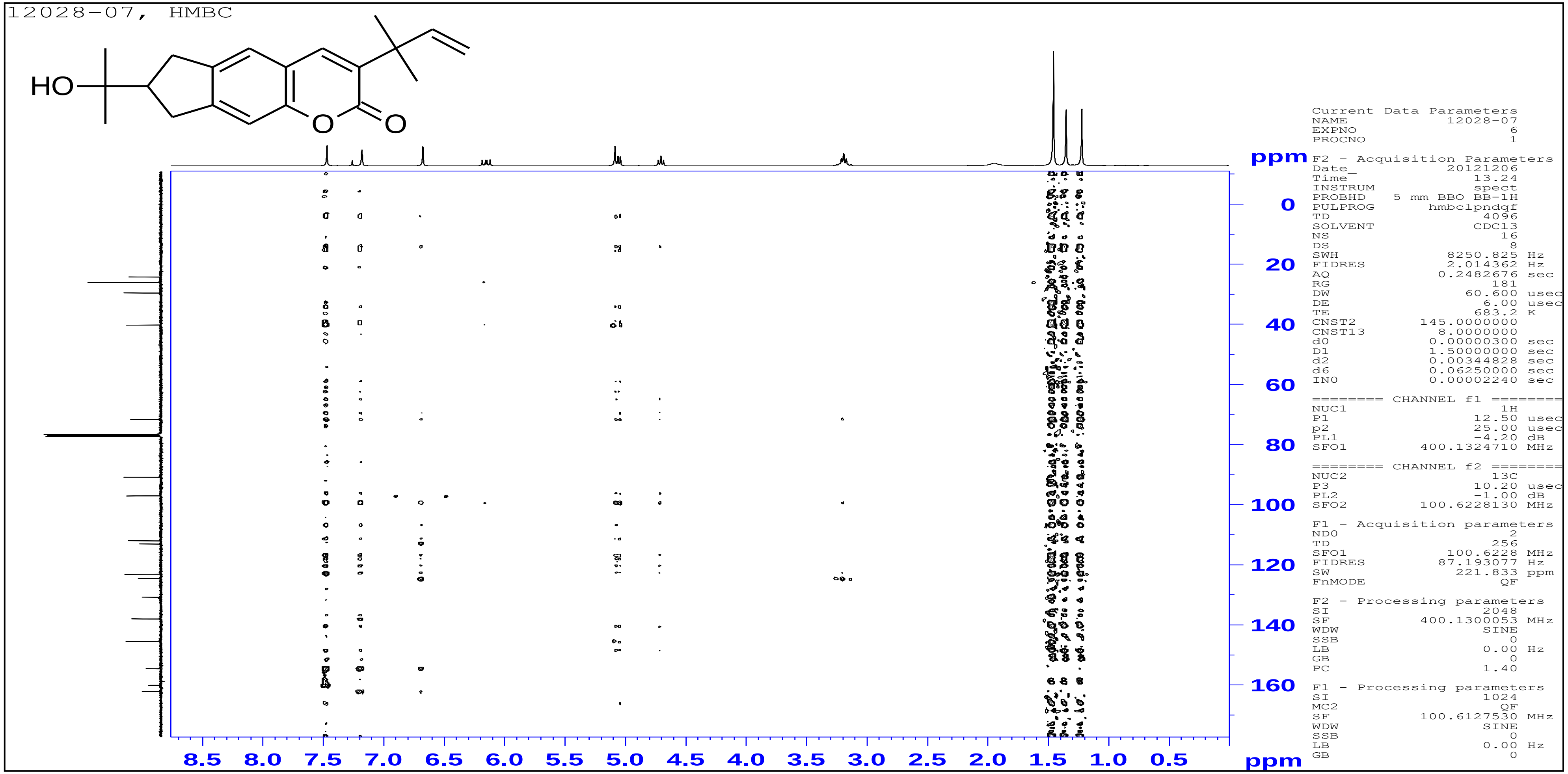

Figure S30. HMBC spectrum of **2**. Obtained by a Bruker Spectrometer Model Advance DPX400, 9.4 Teslas. Operating frequency 400 MHz. ($CDCl_3$).

Spectroscopic analysis of Chalepin **2**

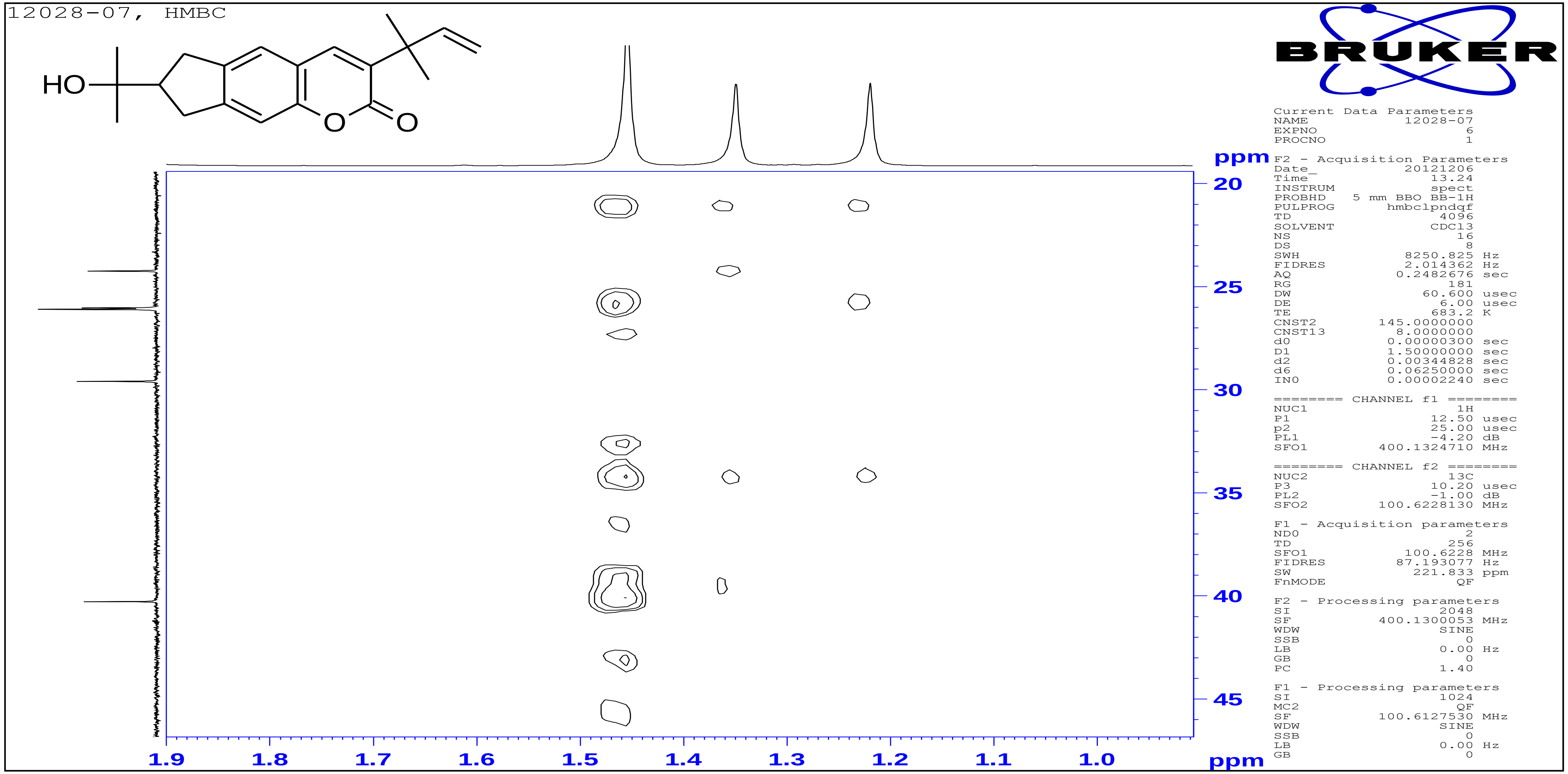

Figure S31. Expansion in the area ^{1}H: 0.9-1.9 ppm, ^{13}C: 20-45 ppm. HMBC spectrum of **2**. Obtained by a Bruker Spectrometer Model Advance DPX400, 9.4 Teslas. Operating frequency 400 MHz. ($CDCl_3$).

Spectroscopic analysis of Chalepin **2**

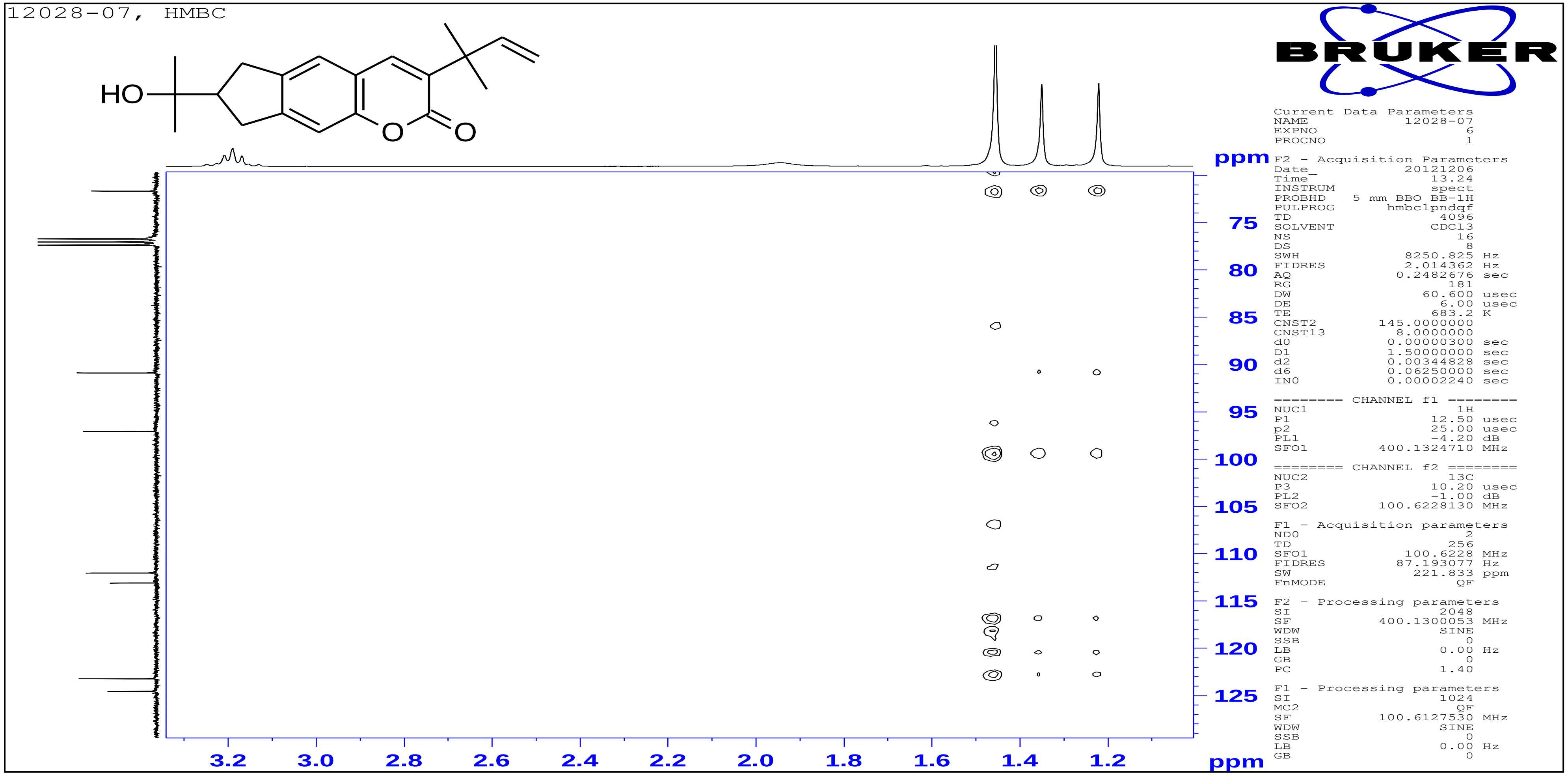

Figure S32. Expansion in the area ^{1}H: 1.0-3.4 ppm, ^{13}C: 70-130 ppm. HMBC spectrum of **2**. Obtained by a Bruker Spectrometer Model Advance DPX400, 9.4 Teslas. Operating frequency 400 MHz. ($CDCl_3$).

Spectroscopic analysis of Chalepin **2**

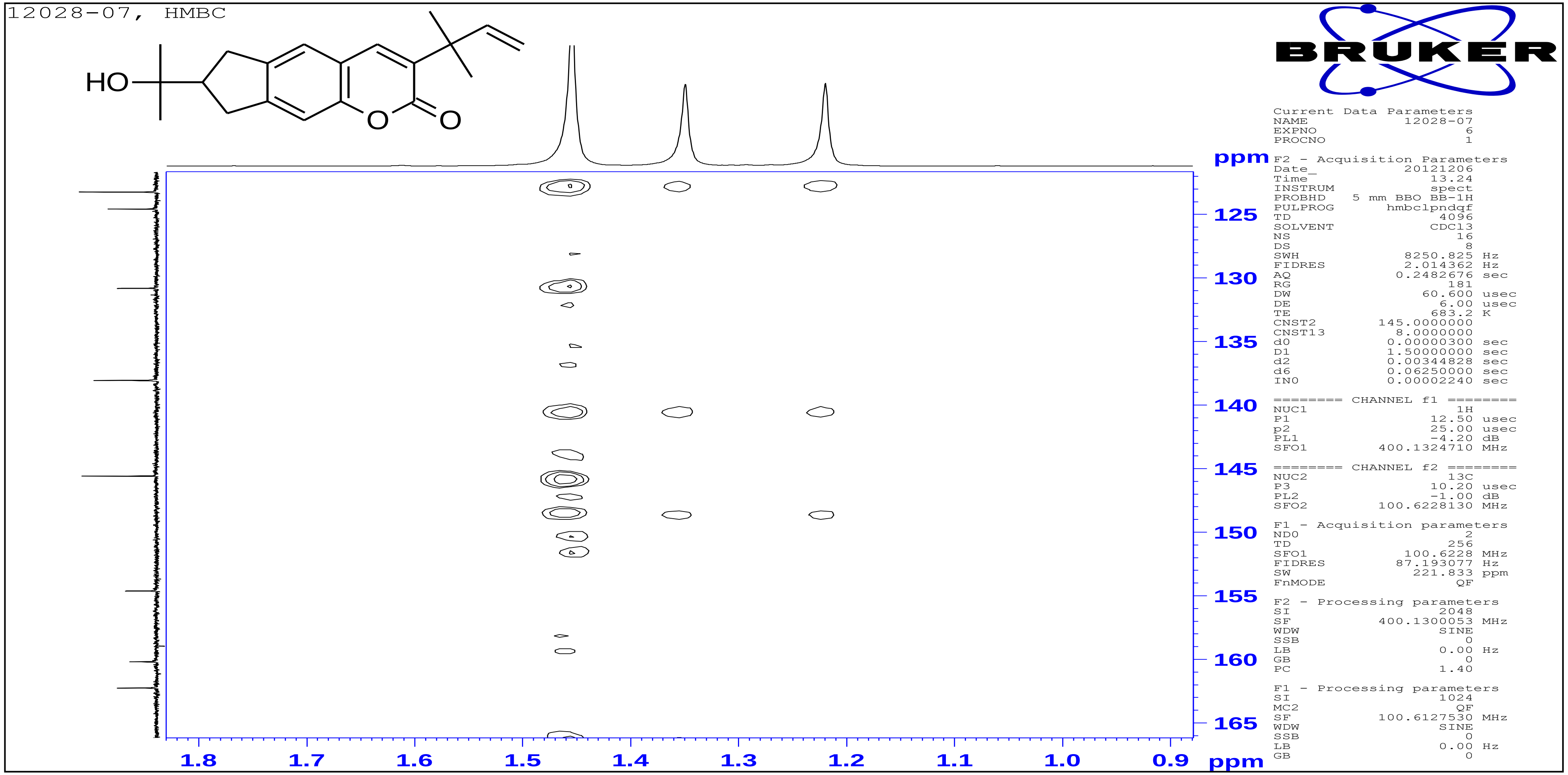

Figure S33. Expansion in the area ^{1}H: 0.9-1.8 ppm, ^{13}C: 120-185 ppm. HMBC spectrum of **2**. Obtained by a Bruker Spectrometer Model Advance DPX400, 9.4 Teslas. Operating frequency 400 MHz. ($CDCl_3$).

Spectroscopic analysis of Chalepin **2**

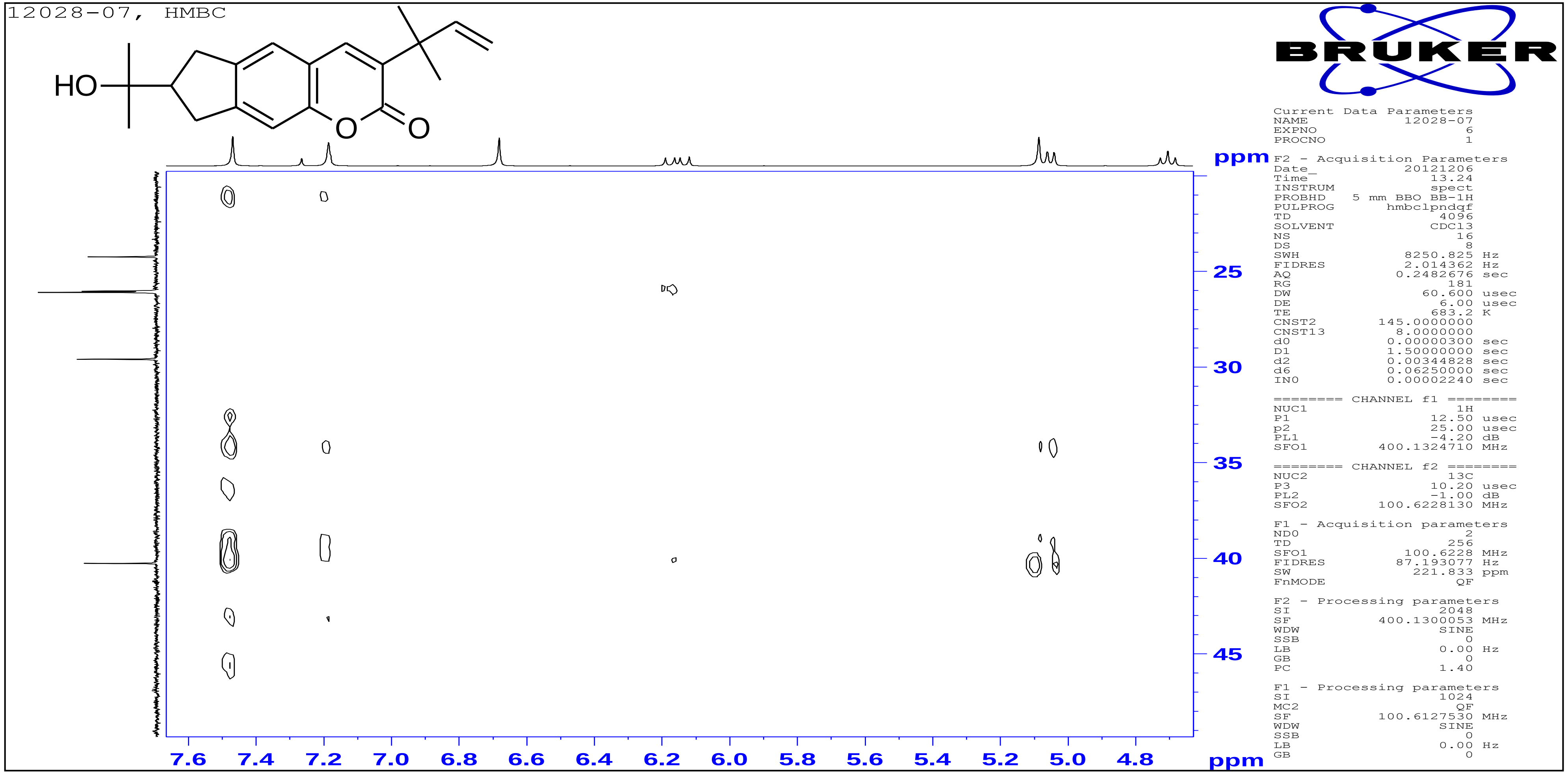

Figure S34. Expansion in the area ^{1}H: 4.6-7.6 ppm, ^{13}C: 20-50 ppm. HMBC spectrum of **2**. Obtained by a Bruker Spectrometer Model Advance DPX400, 9.4 Teslas. Operating frequency 400 MHz. ($CDCl_3$).

Spectroscopic analysis of Chalepin **2**

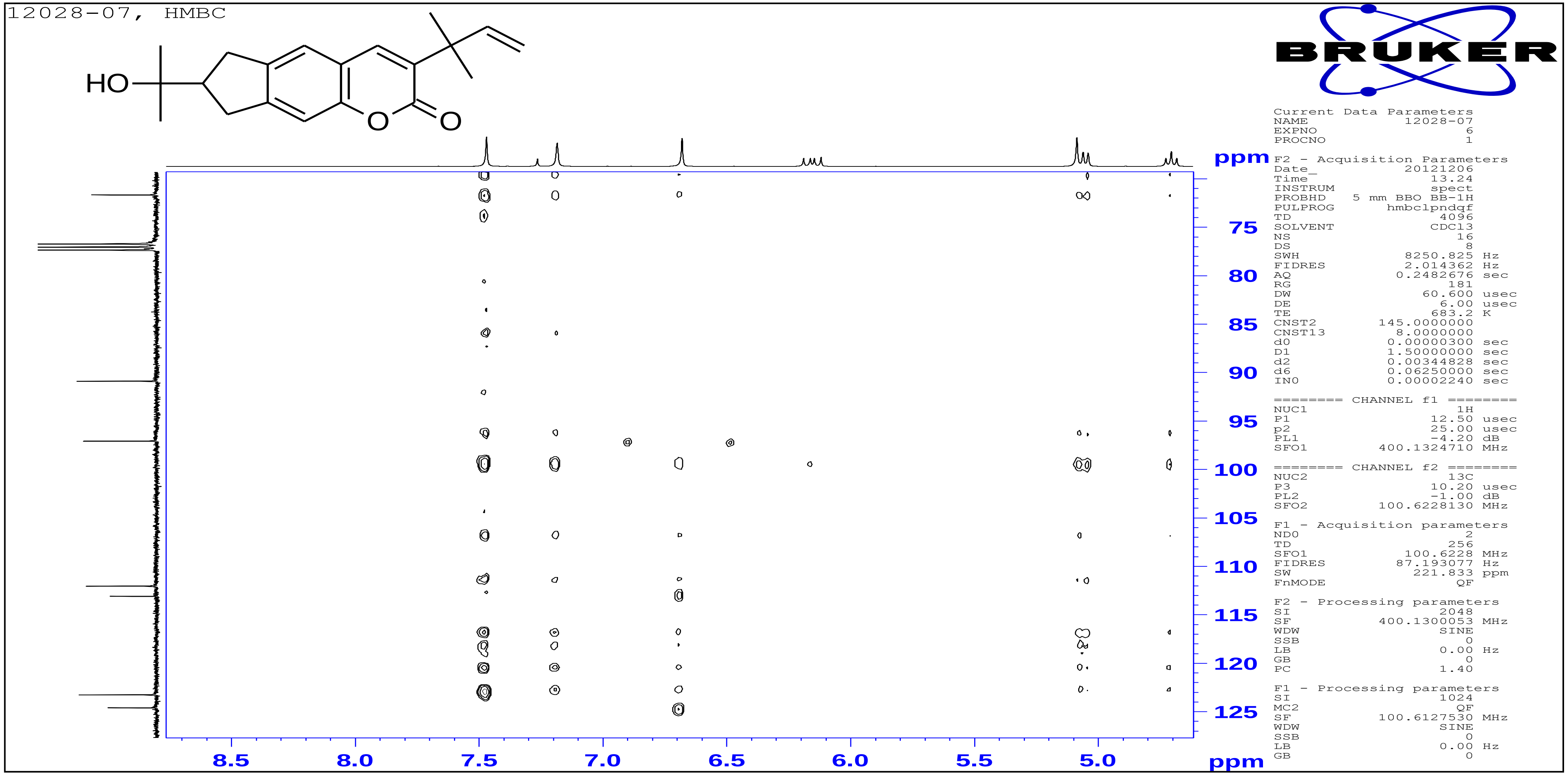

Figure S35. Expansion in the area ^{1}H: 4.6-8.5 ppm, ^{13}C: 70-128 ppm. HMBC spectrum of **2**. Obtained by a Bruker Spectrometer Model Advance DPX400, 9.4 Teslas. Operating frequency 400 MHz. ($CDCl_3$).

Spectroscopic analysis of Chalepin **2**

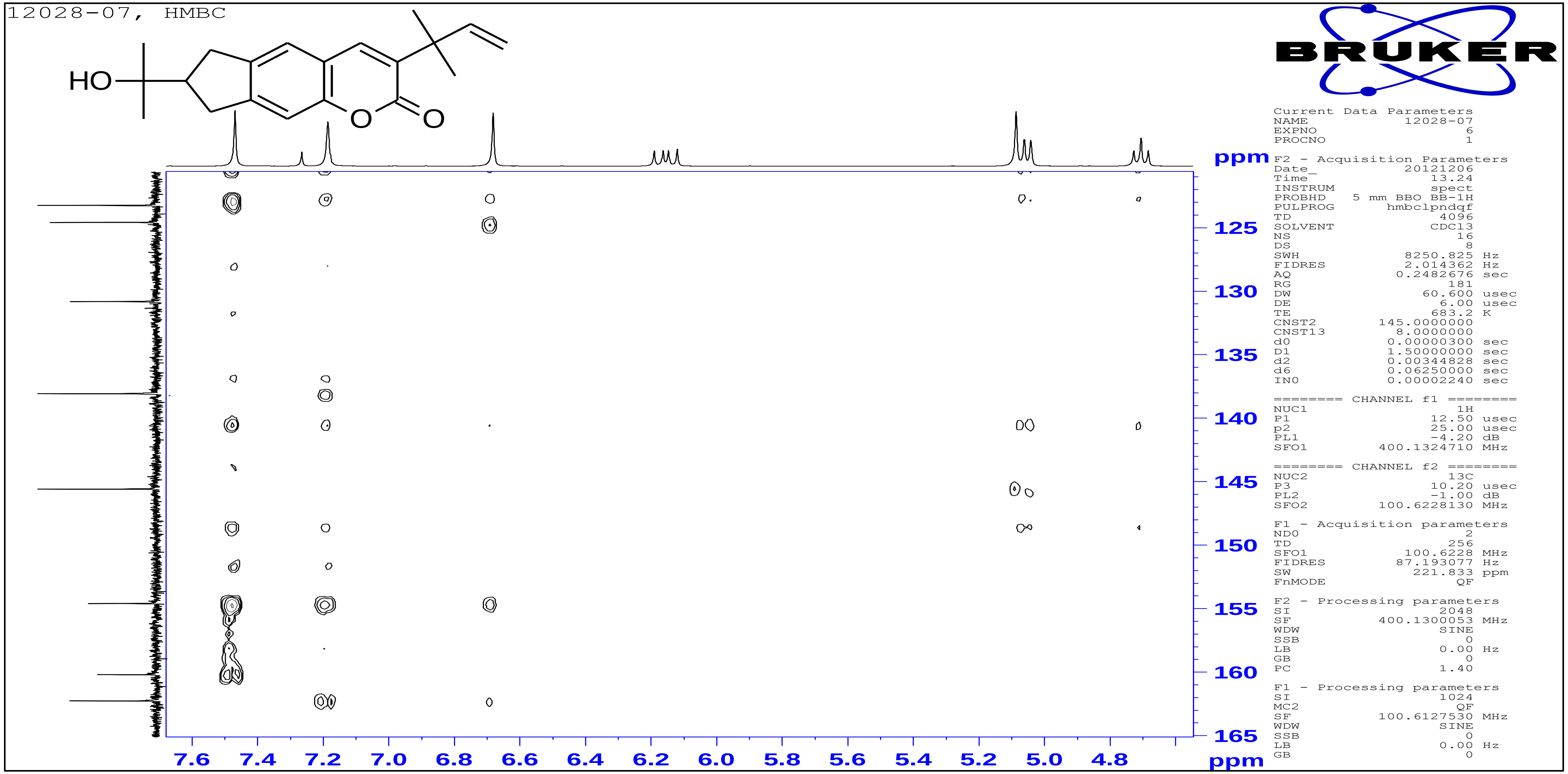

Figure S36. Expansion in the area ^{1}H: 4.6-7.6 ppm, ^{13}C: 120-165 ppm. HMBC spectrum of **2**. Obtained by a Bruker Spectrometer Model Advance DPX400, 9.4 Teslas. Operating frequency 400 MHz. ($CDCl_3$).

Rutamarin (**1**) was recovered as a white amorphous solid (m. p. 104 °C; Lit. 107–109 °C; [1]. Spectroscopy data of this compound corresponded to a previous compound reported as rutamarin, **1** [1]. ^{1}H-NMR ($CDCl_3$, 400.13 MHz) δ: 7.47 (1H, s, H-4), 7.18 (1H, s, H-5), 6.70 (1H, s, H-8), 6.16 (1H, dd, *J* = 17.20, 10.86, 10.85 Hz, H-2"), 5.08 (2H, m, H-3''), 5.04 (1H, m, H-2'), 3.19 (2H, m, H-1'), 1.98 (3H, s, $COCH_3$), 1.55 (3H, s, H-5'), 1.50 (3H, s, H-4'), 1.46 (6H, s, H-4'', H-5''). ^{13}C-NMR ($CDCl_3$, 100.61 MHz) δ: 170.3 (C=O), 162.4 (C-7), 160.2 (C-2), 154.7 (C-8a), 145.6 (C-2"), 138.0 (C-4), 130.9 (C-3), 123.9 (C-6), 123.1 (C-5), 113.1 (C-4a), 112.1 (C-3''), 97.1 (C-8), 88.3 (C-2'), 82.2 (C-3'), 49.3 (C-1''), 29.7 (C-1'), 26.1 (C-4'', C-5''), 22.3 ($COCH_3$), 21.9 (C-5'), 21.0 (C-4').

Chalepin (**2**) was obtained as a yellow amorphous solid (m. p. 125–126 °C; Lit. 117–118 °C) [2]. Spectroscopy data of this compound corresponded to a previous compound reported as chalepin, **2** [3]. ^{1}H-RMN ($CDCl_3$, 400.13 MHz) δ: 7.47 (1H, s, H-4), 7.18 (1H, s, H-5), 6.8 (1H, s, H-8), 6.15 (1H, dd, *J* = 10.88, 10.86, 17.20, H-2"), 5.07 (2H, m, H-3"), 4.70 (1H, t, H-2'), 3.44 (2H, m, H-1'), 1.94 (1H, s, -OH), 1.45 (6H, s, H-4", H-5"), 1.35 (3H, s, H-4'), 1.22 (3H, s, H-5'). ^{13}C-NMR ($CDCl_3$, 100.61 MHz) δ: 162.3 (C-7), 160.2 (C-2), 154.6 (C-8a), 145.6 (C-2"), 138.1 (C-4), 130.8 (C-3), 124.6 (C-4a), 123.2 (C-5), 113.11 (C-6), 112.1 (C-3"), 97.1 (C-8), 90.9 (C-2'), 71.7 (C-3'), 40.3 (C-1"), 29.6 (C-1'), 26.1 (C-4'',5''), 26.0 (C-4'), 24.2 (C-5').

1. Wu, T.S.; Shi, L.S.; Wang, J.J.; Iou, S.C.; Chang, H.C.; Chen, Y.P.; Kuo, Y.H.; Chang, Y.L.; Teng, C.M. Cytotoxic and antiplatelet aggregation principles of Ruta graveolens. *J. Chinese Chem. Soc.* **2003**, *50*, 171–178.
2. Ezmirly, S.T.; Wilson, S.R. Saudi Arabian medicinal plants I: Ruta Chalepensis. *J. Chem.* **1980**, *2*, 55–57.
3. Richardson, J.S.M.; Sethi, G.; Lee, G.S.; Malek, S.N.A. Chalepin: Isolated from Ruta angustifolia L. Pers induces mitochondrial mediated apoptosis in lung carcinoma cells. *BMC Complement. Altern. Med.* **2016**, *16:389*, 1–27.

Figure S37. NMR Data of Rutamarin (**1**) and Chalepin (**2**)

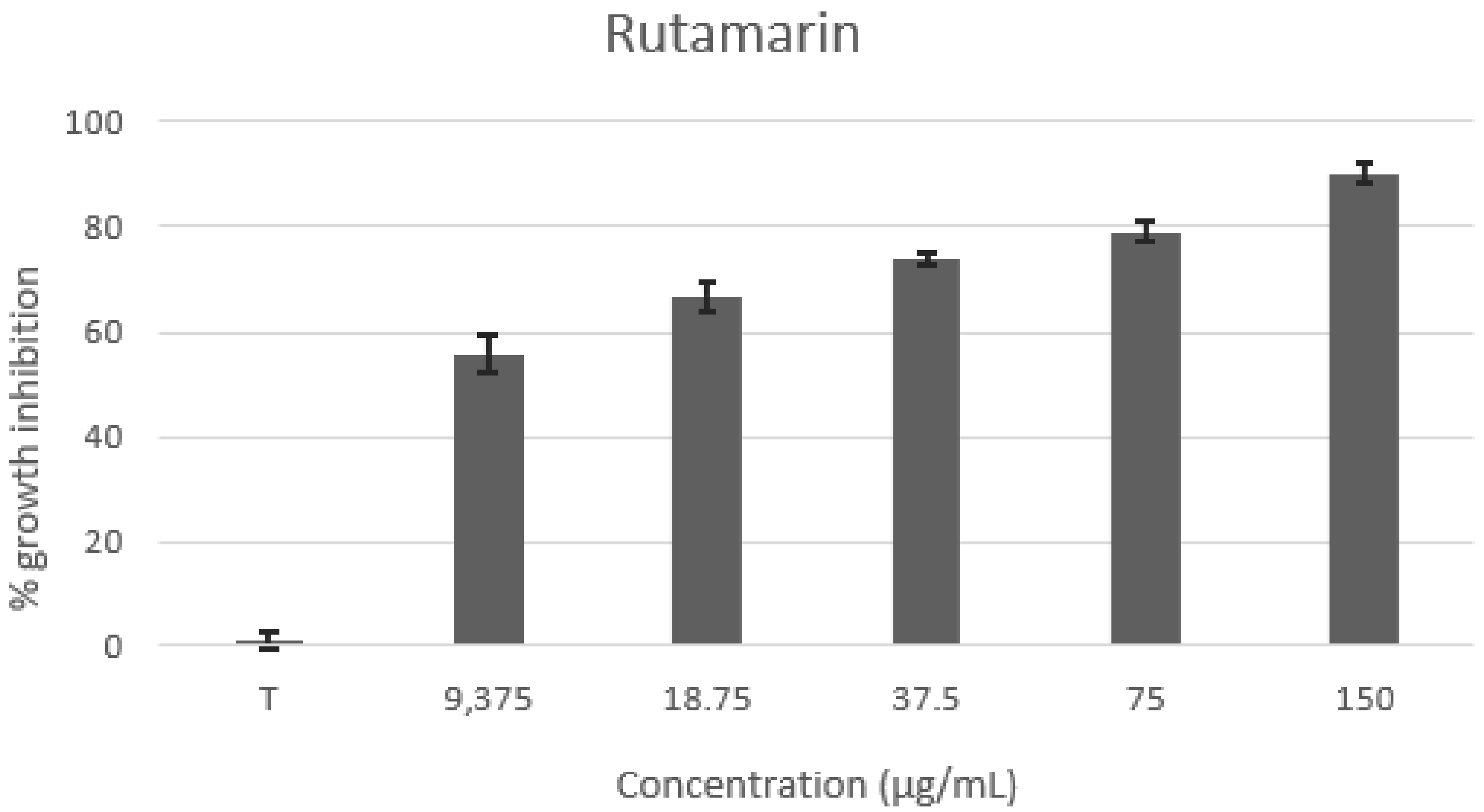

Figure S38. Antiprotozoal activity of Rutamarin (**1**) against trophozoite of *E. histolytica.* T = negative control consisting of culture medium and parasites.

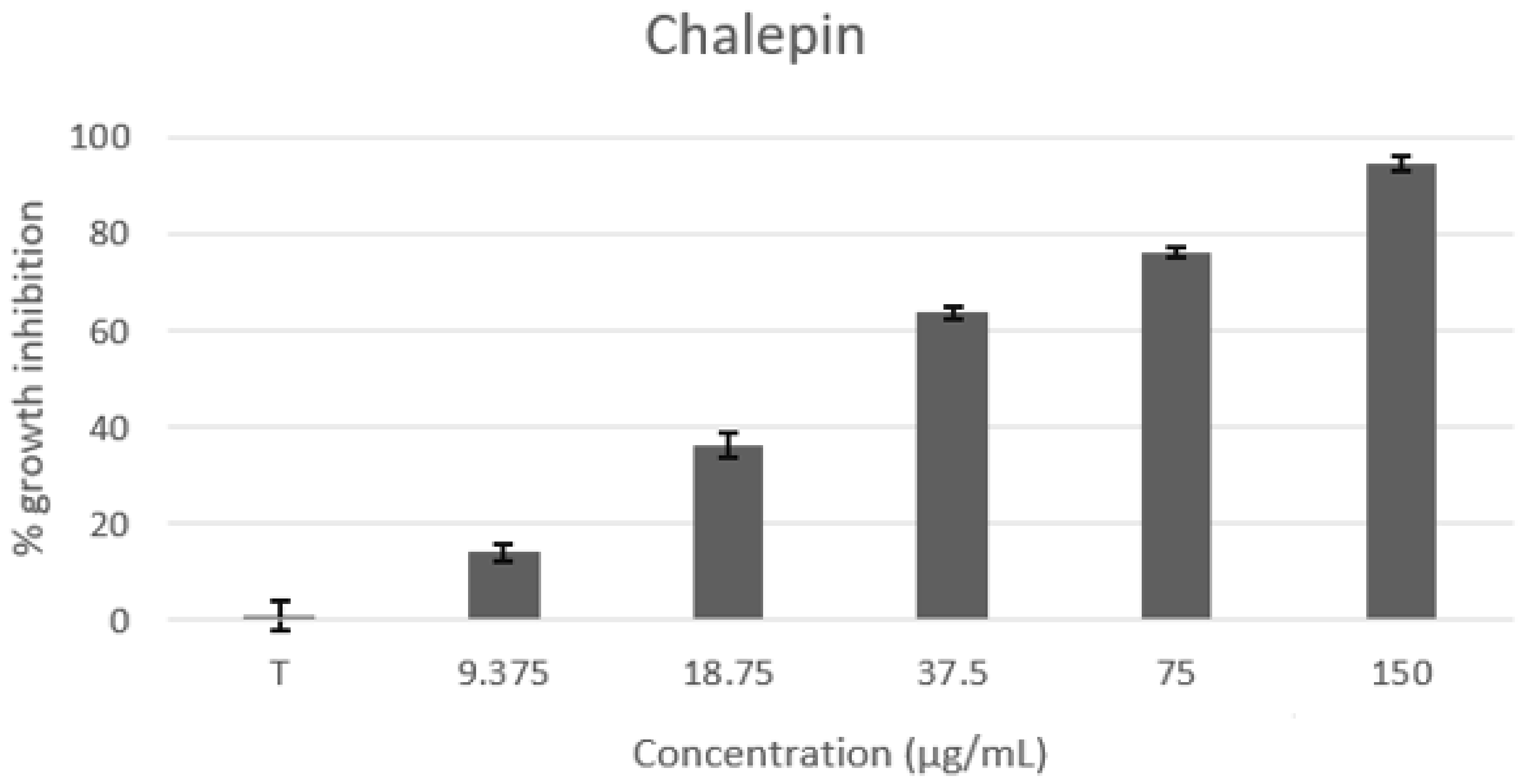

Figure S39. Antiprotozoal activity of Chalepin) (**2**) against trophozoite of *E. histolytica.* T = negative control consisting of culture medium and parasites.

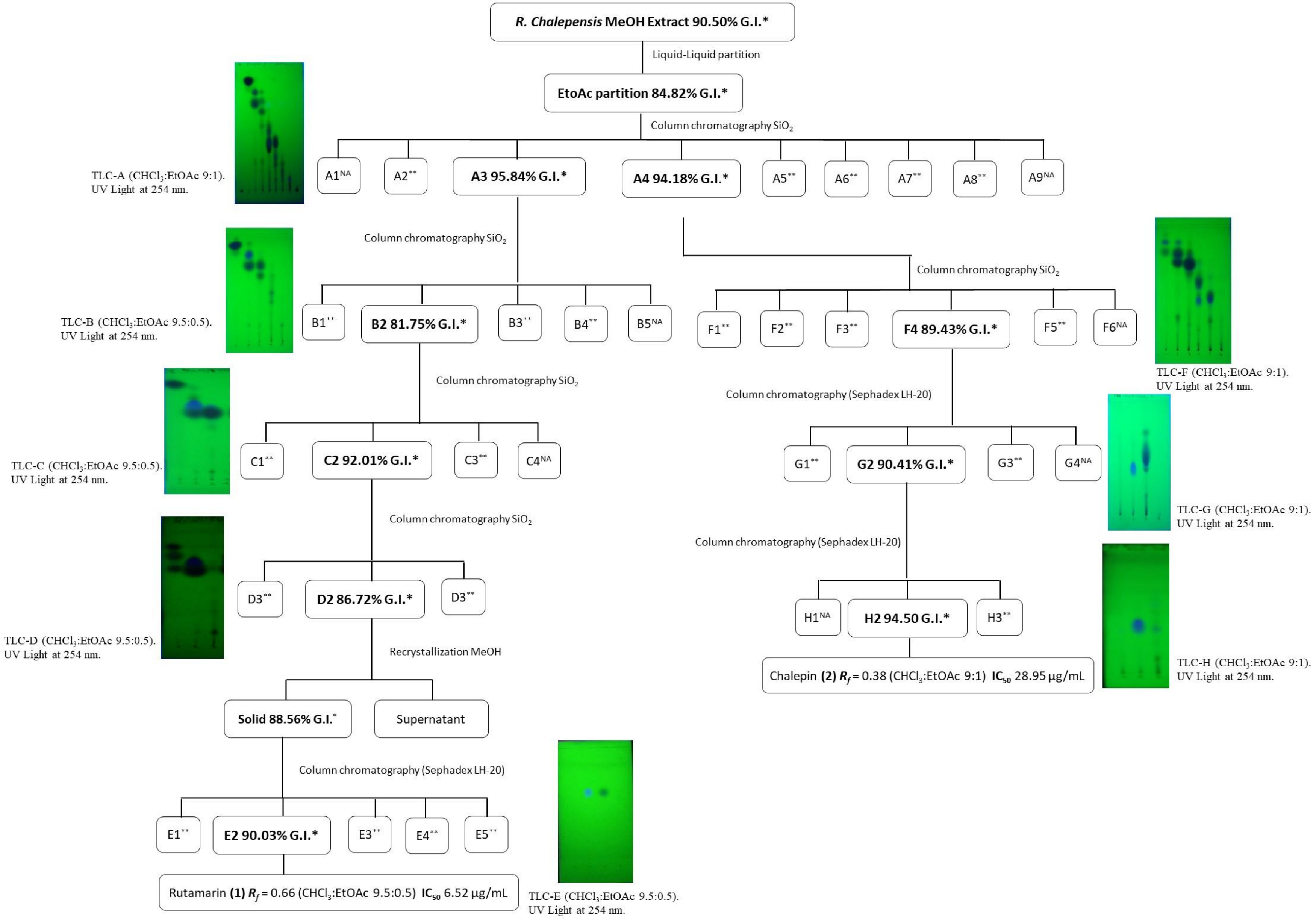

* Growth inhibition on *E. histolytica* at 150 µg/mL. **Growth inhibition on *E. histolytica* less than 50% at 150 µg/mL. [NA] No amebicide activity

Figure S40. General scheme for the bioguided isolation of compounds with antiamoebic activity from *Ruta chalepensis*
